# Comparative effectiveness of non-pharmacological interventions for depression and anxiety in chronic low back pain: a Bayesian network meta-analysis of randomized controlled trials

**DOI:** 10.3389/fpubh.2026.1765762

**Published:** 2026-04-20

**Authors:** Lei Chen, Li Zeng, Yi-tian Lai, Wu Li, Jiang-shan Li

**Affiliations:** Department of Tuina, School of Acupuncture-Moxibustion and Tuina, Hunan University of Chinese Medicine, Changsha, Hunan, China

**Keywords:** anxiety, Bayesian network meta-analysis, chronic low back pain, depression, non-pharmacological interventions

## Abstract

**Background:**

Chronic low back pain (CLBP) is often accompanied by anxiety and depression, which hinders clinical treatment. Therefore, this study conducts a Bayesian network meta-analysis to compare the effectiveness of different non-drug interventions in relieving pain and related emotional symptoms in CLBP patients.

**Methods:**

This review was prospectively registered in PROSPERO (CRD420251066414). As of June 2025, PubMed, Embase, Cochrane Library, Web of Science, CINAHL, Scopus, CNKI, and Wanfang databases have been searched to collect randomized controlled trials concerning non-pharmacological interventions for CLBP. The main outcomes are pain, anxiety, and depression. The scoring directions of all outcome scales were consistent. To account for differences in measurement tools, effect sizes are expressed as standardized mean differences (SMD), estimated using a Bayesian random-effects model based on change scores, and reported with 95% credible intervals (CrIs), adjusted using Hedges' g. Comprehensive sensitivity analysis, subgroup analysis, and regression analysis were carried out to explore the source of heterogeneity and the factors affecting the treatment results.

**Results:**

Twenty-four trials were ultimately included (*n* = 1828), involving 9 non-pharmacological interventions. The analysis of the results shows that mind-body exercises (MBE) demonstrated favorable effects for pain relief [SMD: −1.55, 95% CrI (−2.50, −0.59)] and depression and anxiety reduction [SMD: −1.14, 95% CrI (−1.74, −0.54), SMD: −1.38, 95% CrI (−1.95, −0.81)]. Structured exercise (SE) was also associated with relief in pain [SMD: −1.02, 95% CrI (−1.94, −0.09)], depression, and anxiety [SMD: −0.77, 95% CrI (−1.35, −0.19), SMD: −0.69, 95% CrI (−1.25, −0.14)]. Integrated rehabilitation therapy (IR) showed a potential benefit for improving depression [SMD: −0.74, 95% CrI (−1.28, −0.18)]. Considerable heterogeneity was observed across all outcome measures (pain: 86.24%; depression: 70.64%; anxiety: 66.08%). Additional subgroup interaction analyses did not identify any factors influencing treatment outcomes. Meta-regression suggested that the number of interventions may influence pain, whilst the type of intervention may influence anxiety.

**Conclusion:**

MBE and SE may help alleviate pain, anxiety, and depression among CLBP individuals, whilst IR may have a modest beneficial impact on depression. Given the high heterogeneity across different outcome measures, these conclusions should be evaluated with caution, and further high-quality research is required to substantiate them.

**Systematic Review Registration:**

https://www.crd.york.ac.uk/prospero/display_record.php?ID=CRD420251066414, PROSPERO, CRD420251066414.

## 1 Introduction

Chronic low back pain (CLBP) refers to repeated pain that lasts for at least 3 months (1), which is one of the main causes of disability worldwide. In 2020, about 619 million people worldwide suffered from CLBP, and this figure is expected to increase by 36.4% by 2050 (2). In addition to typical persistent pain, CLBP also affects the patient's daily life (3) and income. Research by Parthan et al. (4) found that the disfunction caused by CLBP will reduce the patient's income, and pain-related symptoms will also hinder treatment. Recent evidence shows that CLBP and diseases such as anxiety and depression have common pathophysiological mechanisms, including neuroinflammation, central sensitization, and disruption of the dopaminergic system (5–7). There is a two-way relationship between pain and emotion. Negative emotions will aggravate pain perception, and pain will cause emotional distress, thus forming a vicious circle (8, 9). The above mechanism also explains the high incidence of mental disorders in older adults patients with CLBP (3, 10, 11).

Traditionally, the drug treatment of CLBP depends on opioids and non-steroidal anti-inflammatory drugs. These drugs can relieve pain in the short term, but long-term use will bring risks such as gastrointestinal complications and opioid dependence (12, 13). Therefore, the World Health Organization recommends non-pharmacological interventions as a first-line treatment plan (14). Because these methods are safer than drugs, they can effectively relieve pain, anxiety, and depression, and improve physical function (15). However, there are still shortcomings in the relevant research on non-pharmacological interventions. Briggs et al. (16) noted a lack of comparative research on various non-pharmacological interventions. Most trials focused on the improvement of pain and functional activity, ignoring the measurement of psychological outcomes.

In order to make up for the above gaps and assess the multidimensional impact of interventions on the physical and psychological outcomes of people with CLBP, this study uses network meta-analysis (NMA) to compare the effects of nine non-pharmacological therapies in relieving pain, anxiety, and depression in patients with CLBP, thereby enabling consistent comparisons of intervention effects within the same study population. Unlike traditional meta-analysis, NMA can compare multiple interventions at the same time and sort their efficacy. This method aims to optimize the treatment plan of CLBP and its related emotions for clinicians. However, as the analysis included only trials reporting three outcomes, this may have resulted in the exclusion of some rigorous studies focusing on a single outcome, thereby limiting the representativeness of the evidence to some extent. Consequently, the conclusions of this study may be more applicable to studies assessing physical and psychological symptoms.

## 2 Methods

This NMA has been registered with PROSPERO (CRD420251066414) and is carried out in strict accordance with the PRISMA guidelines. The study included a randomized controlled trial (RCT) of non-drug interventions for the treatment of pain, depression, and anxiety symptoms in patients with CLBP.

### 2.1 Search strategy and study selection

PubMed, Embase, Cochrane Library, Web of Science, CINAHL, Scopus, CNKI, and Wanfang databases have been comprehensively retrieved, covering English and Chinese articles published from the establishment of the database to June 2025. The search strategy integrated Medical Subject Headings (MeSH) with relevant free-text terms, including ‘chronic low back pain', ‘acupuncture', ‘massage', ‘yoga', ‘cognitive behavioral therapy', ‘anxiety', and ‘depression', along with related synonyms. Details of the full search strategy are provided in Supplementary Table S1.

### 2.2 Inclusion and exclusion criteria

This review conformed to the Participants, Intervention, Comparison, Outcomes, and Study Design (PICOS) criteria.

Inclusion criteria were as follows: (i) participants were adults aged 18 years or older diagnosed with CLBP of at least 12-week duration; (ii) the experimental group received one or more non-pharmacological interventions, such as massage therapy, acupuncture, yoga, exercise therapy, or cognitive therapy, whereas the control group was assigned a placebo or sham intervention, standard non-pharmacological care, or another non-pharmacological therapy. the use of stable background medication was permitted as long as it did not constitute the primary intervention; (iii) studies employed a RCT design; (iv) primary outcomes included validated measures of pain [e.g., Visual Analog Scale (VAS), Numerical Rating Scale (NRS)], anxiety [e.g., Hamilton Anxiety Rating Scale (HAMA), Self-Rating Anxiety Scale (SAS), Depression Anxiety Stress Scales-21 (DASS-21)], and depression [e.g., Hamilton Depression Rating Scale (HAMD), Self-Rating Depression Scale (SDS)]. Studies were incorporated only if they reported all three categories of outcomes with complete baseline and post-intervention data (mean, standard deviation, and sample size); (v) the article were published in English or Chinese.

Exclusion criteria were as follows: (i) studies that reported only one or two of the primary outcomes: pain, anxiety, or depression; (ii) studies employing cross-over designs and animal experiments, as well as conference abstracts, study protocols, systematic reviews; (iii) studies with incomplete or missing data or lacking full text.

### 2.3 Data extraction

All articles retrieved through database searches were imported into NoteExpress 4.1.0.10133 for management, with duplicate publications removed. Titles and abstracts were then screened against defined inclusion and exclusion criteria to identify eligible studies. Two reviewers independently extracted data from eligible articles. Information extracted included the first author, year of publication, study location, participant characteristics (age, gender, sample size), details of the intervention and control, and baseline and post-intervention outcome data (mean and standard deviation). Any discrepancies during the process were resolved through discussion with a third reviewer.

For analytic convenience, the non-pharmacological interventions explored in the encompassed studies were classified as the following groups, according to their primary therapeutic objectives: cognitive behavioral therapy (CBT), education with behavioral activation (EBA), relaxation and psychological education (RPE), mind–body exercise (MBE), structured exercise (SE), functional and targeted exercise (FTE), passive physical therapy (PPT), digital and biofeedback therapy (DBT), and integrated rehabilitation (IR). An elaborate description of each intervention category is provided in Table 1.

**Table 1:** Introduction to the control group and intervention measures.

Intervention category	Abbreviation	Specific examples	Duration and frequency of treatment	Description
Control	Con	Wait-list, usual care, placebo	1–13 weeks; 1–30 sessions	Includes standard care, health education, wait-list, or placebo groups
Cognitive behavioral therapy	CBT	Cognitive behavioral therapy	4–9 weeks; 8–15 sessions	A psychological approach that aims to alleviate symptoms by changing thinking and behavior patterns
Education with behavioral activation	EBA	Therapeutic patient education	5–9 weeks; 7–15 sessions	To enhance patients' understanding of the biology of pain and self-management strategies
Relaxation and psychological education	RPE	binaural beats audio and music therapy	2–4 weeks; 1–52 sessions	Utilizes relaxation techniques and psychological knowledge to reduce symptoms
Mind body exercise	MBE	Yoga, Yijinjing	1–12 weeks; 12–68 sessions	Exercises that integrate physical activity with mental regulation
Structured exercise	SE	Resistance training, stretching, general strength training	4–12 weeks; 3–60 sessions	Physical exercises with a clear plan and specific instructions
Functional and targeted exercise	FTE	Motor control exercise (e.g., multifidus training)	1–6 weeks; 6–14 sessions	Strengthening exercises that target specific muscles or functions
Passive physical therapy	PPT	Massage therapy, spinal manipulative therapy	2–6 weeks; 10–28 sessions	Hands-on treatments performed by a therapist where the patient is a passive recipient
Digital and biofeedback therapy	DBT	EMG biofeedback therapy	4–8 weeks; 8 sessions	Utilizes modern technology, such as biofeedback, to help patients with self-regulation
Integrated rehabilitation	IR	Physical therapy + MoodGYM	2–13 weeks; 6–28 sessions	Combines multiple rehabilitation methods, such as manual therapy and exercise, into a single, comprehensive treatment program

### 2.4 Risk of bias and certainty of evidence

The risk of bias and the certainty of evidence for the comprised studies were assessed using the Cochrane Risk of Bias tool (RoB 1.0) and the Confidence in Network Meta-Analysis (CINeMA) framework (17, 18). RoB 1.0 assesses seven areas: random sequence generation, allocation concealment, blinding of participants and personnel, blinding of outcome assessment, incomplete outcome data, selective reporting, and other potential sources of bias. Each area is judged as having a low, unclear, or high risk of bias. CINeMA was applied to appraise the credibility of the network meta-analytic findings across six dimensions: study-level bias, reporting bias, indirectness, imprecision, heterogeneity, and inconsistency (19). The certainty of evidence for each comparison was rated as high, moderate, low, or very low. These assessments were conducted separately by two reviewers, with any discrepancies reconciled through consultation with a third investigator.

### 2.6 Statistical analysis

Bayesian NMA was conducted in R (v4.3.2) using the gemtc package. The analysis was performed using an ARM-based Bayesian hierarchical model. All scales are scored in the same direction (higher scores indicate more severe symptoms). If a study reports multiple follow-up assessments, the time point closest to completion of the therapy is selected for analysis; all outcomes are based on changes score. Given the differences in scoring ranges and measurement characteristics among various scales (e.g. HAMA, SAS, HAMD), the standardized mean difference (SMD) is employed as the primary effect size. Comparability is achieved by standardizing the mean change using the pooled standard deviation, and Hedges' g is used to correct for small-sample bias. For studies that did not report the mean or standard deviation, in accordance with the Cochrane Handbook, confidence intervals, medians and quartiles were used, and the methods proposed by Wan et al. (20) and Luo et al. (21) were employed to estimate the mean and standard deviation. In multi-arm trials, to avoid double-counting of control groups, relevant intervention groups were pooled according to Cochrane methodology, and pooled means and standard deviations were calculated. Analyses used random-effects models with uniform priors on heterogeneity [τ~U(0,5)], as the primary analytical approach. Markov Chain Monte Carlo (MCMC) simulation generated posterior distributions using 4 chains with 200,000 iterations after 20,000 burn-in (thin = 40) to produce posterior SMDs and 95% credible intervals (CrIs) (22). Convergence assessment used the potential scale reduction factor (PSRF; adequate if < 1.1), and model fit was compared via the Deviance Information Criterion (DIC) (23, 24). SMDs were interpreted as small (≈0.2), medium (≈0.5), or large (≈0.8) effects, following established conventions (25), and negative SMDs with CrIs excluding zero indicated beneficial effects. Between-study heterogeneity was quantified using τ and its CrI. To evaluate the consistency assumption, the design-by-treatment *Q*-test and node-splitting were applied. SUCRA scores analyzed intervention rankings. Baseline characteristics were compared using one-way ANOVA for normally distributed variables, or the Kruskal-Wallis *H* test for non-normally distributed data. A *p*-value > 0.05 implies baseline comparability across groups.

Adjusted funnel plots were utilized to assess potential publication bias in the NMA; differences between treatments were eliminated—i.e., centralized—by subtracting the network's pooled estimate from each study's effect size. This method presumes that study effect sizes are independent, that standard errors reflect study precision, and that effect sizes from small-sample studies may be systematically overestimated in the presence of publication bias. In addition to visual inspection, the centralized effect sizes were subjected to adjusted Egger's regression and Begg's rank correlation tests. The potential impact of missing studies was assessed via pruning and imputation analyses. Sensitivity analyses began by examining the distribution of effect sizes using box plots and scatter plots to identify outliers, whilst calculating the Bayesian leverage and Cook's distance for each study; high-impact studies were defined as those with a Cook's distance exceeding the 75th percentile of all studies. Further analysis examined the relationship between sample size and effect size to identify studies deviating significantly from the network-weighted mean (26, 27), followed by the exclusion of outlier studies using a stepwise elimination method. Additionally, the analysis was rerun using a fixed-effects model to assess the robustness of the SUCRA rankings and the overall effect estimates. In addition, in order to explore the source of heterogeneity, the interaction analysis of subgroup stratification was carried out according to age, research quality, and the number, frequency and time of interventions, baseline pain levels, and the degree of therapist involvement. At the same time, the regression analysis with these factors as continuous predictors is supplemented.

## 3 Results

### 3.1 Literature search

**Figure 1:**
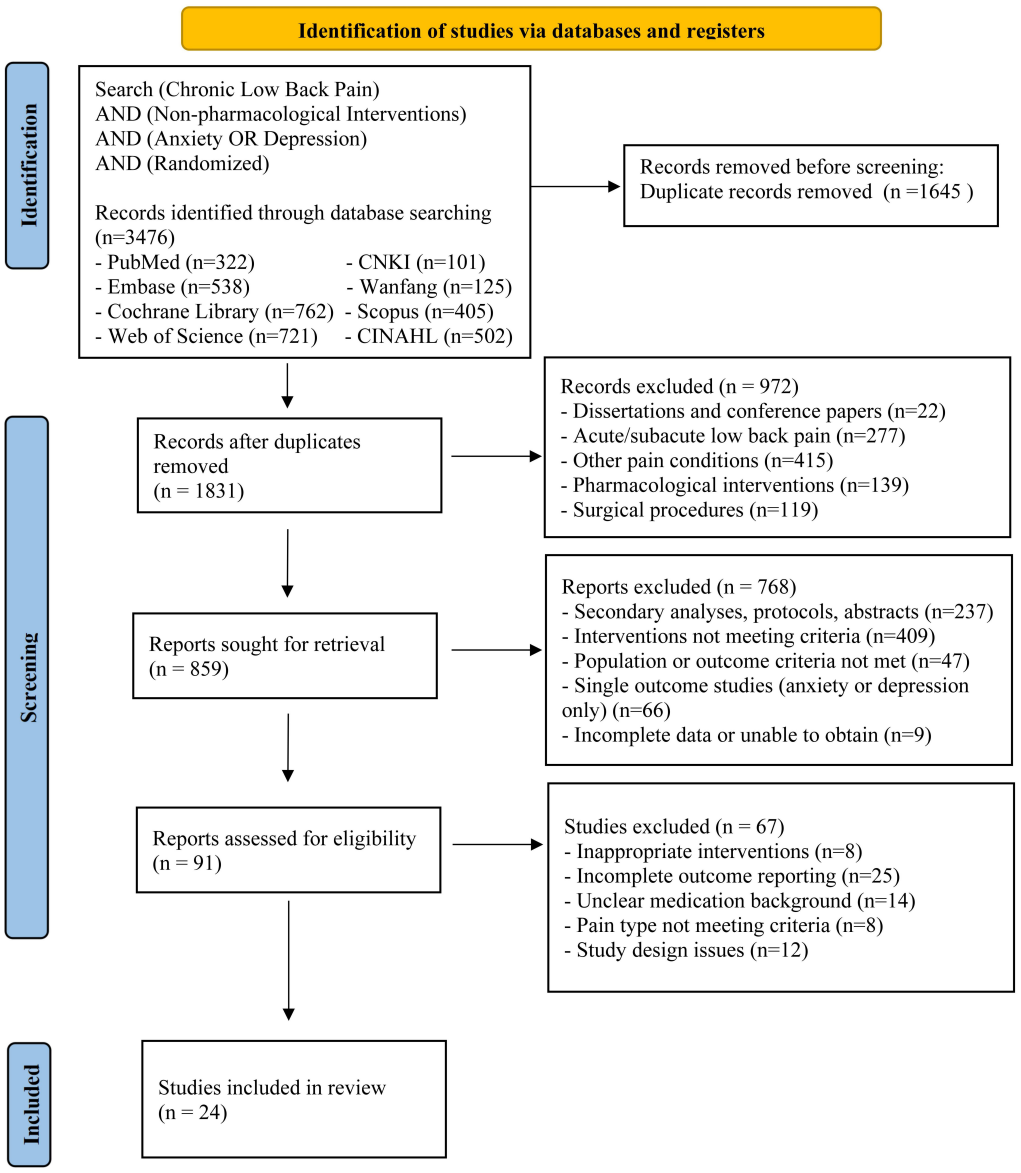
PRISMA flow diagram of the study process.

Database searches identified 3,476 articles. After removing duplicates, 1,831 records underwent title and abstract screening, yielding 91 potentially eligible studies. Following full-text review, 24 studies met the inclusion criteria (28–51). Figure 1 shows the PRISMA screening flow and reasons for exclusion.

### 3.2 Studies characteristics

Across 24 studies (28–51), involving a total of 1,828 participants, nine categories of non-pharmacological therapies were examined in addition to control groups. The interventions comprised CBT (28, 35, 38, 40), EBA (28, 29, 37, 51), RPE (28, 42, 44), MBE (30, 31, 33, 41, 45), SE (30, 31, 50, 51), FTE (29, 34, 41, 49), PPT (32, 39, 43, 47–49), DBT (40, 46), and IR (32, 34–37, 43, 49). All included studies reported outcome measures related to pain, anxiety, and depression. Detailed information on the 24 studies (including basic study details, participant information, interventions, and individual outcome measures) is presented in Supplementary Tables S2, S3.

### 3.3 Risk of bias

**Figure 2:**
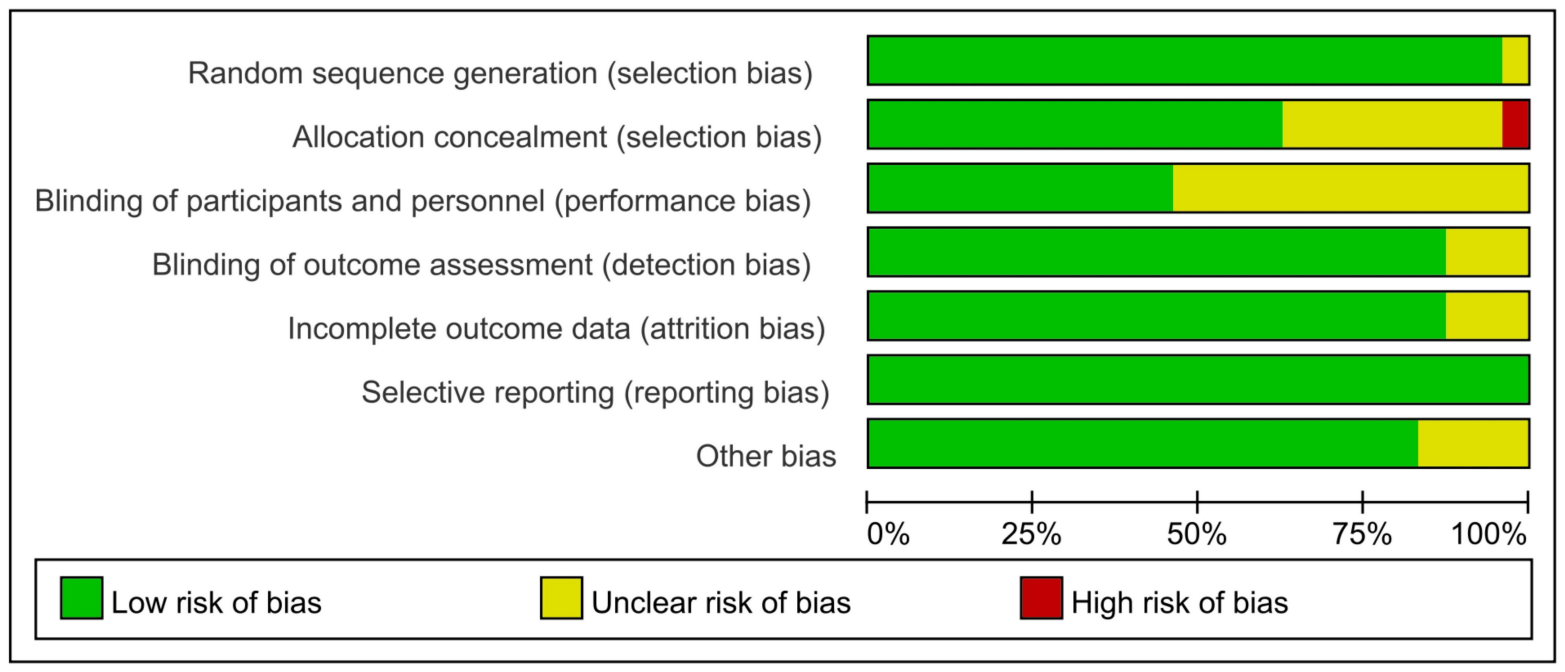
Risk of bias graph.

**Figure 3:**
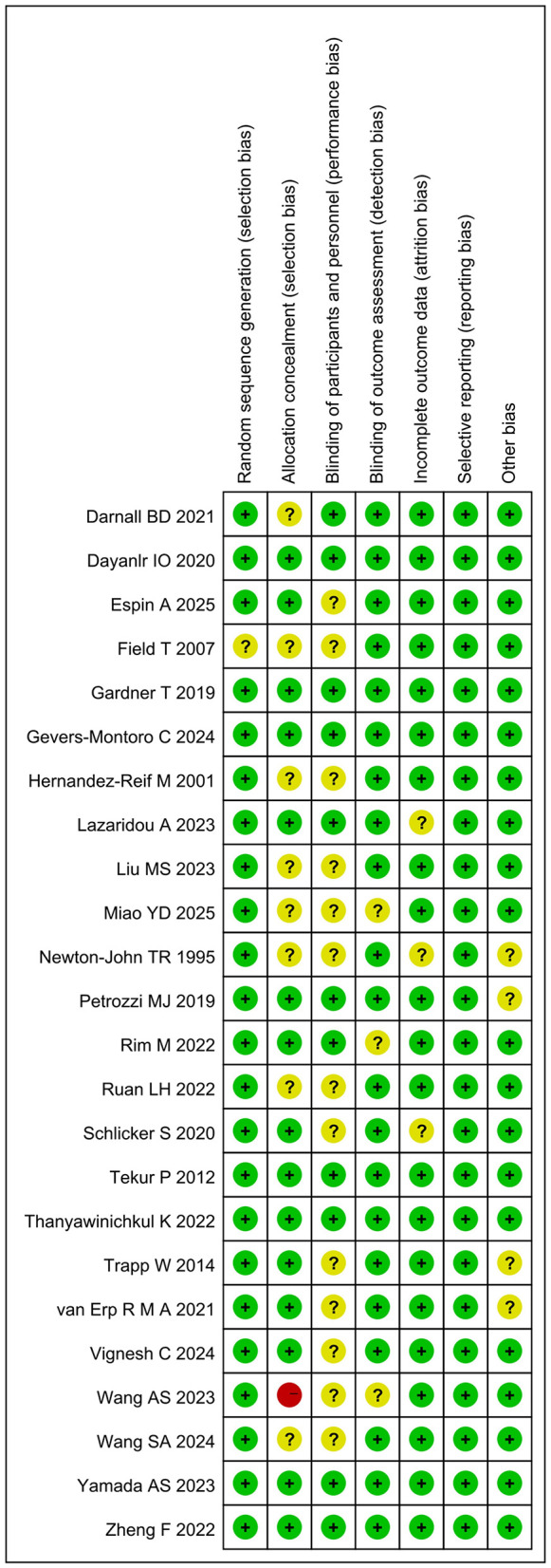
Risk of bias summary.

In 23 of the 24 studies (96%), randomization sequences were generated, indicating minimal risk of selection bias in these investigations. Fifteen trials (63%) reported allocation concealment. Additionally, 11 studies employed blinding for participants or researchers, while 21 studies (88%) implemented blinding for outcome assessors. Moreover, outcome data were largely complete, with 21 studies (88%) showing low attrition-related bias. All included trials demonstrated adherence to prespecified reporting, without risk of selective outcome reporting. The overall assessment of risk of bias is summarized in Figures 2, 3.

### 3.4 Certainty of evidence assessment

The CINeMA evaluation, summarized in Supplementary Figures S1–S3, examined risk of bias, reporting bias, indirectness, imprecision, heterogeneity, and inconsistency for all outcomes across the nine intervention categories. On the whole, the certainty of evidence exhibited a range of very low to moderate for pain, anxiety, and depression.

### 3.5 NMA results

A NMA was conducted across the 24 included trials to evaluate pain, anxiety, and depressive symptoms. All models demonstrated satisfactory convergence, with PSRF values remaining below 1.1 and effective sample sizes indicating stable estimates. Comparison of model fit showed consistently lower DIC values for the random-effects models than for the fixed-effects counterparts, supporting the use of the random-effects framework in deriving pooled effect estimates. Nonetheless, there was considerable heterogeneity observed among the outcomes: τ for pain was 0.801 (95% CI: 0.549–1.172), *I*^2^: 86.24%, τ for depression was 0.462 (95% CI: 0.286–0.714), *I*^2^: 70.64%, and τ for anxiety was 0.440 (95% CI: 0.260–0.696), *I*^2^: 66.08%. Despite the presence of heterogeneity, the design-by-treatment Q-tests under the random-effects framework indicated good global consistency for three outcomes (pain: *p* = 0.738; depression: *p* = 0.972; anxiety: *p* = 0.562; all *p* ≥ 0.05).

#### 3.5.1 Baseline characteristics

The transitivity hypothesis was assessed by comparing participants' mean age, baseline pain intensity, intervention frequency, number of sessions, duration, and therapist involvement. The distributions of each potential effect-modifying variable are shown in Supplementary Figure S4. The results indicated that the distributions of the variables overlapped extensively, with no apparent gaps in distribution. Statistical analysis (Table 2) revealed that most clinical variables were comparable between intervention groups (*P* > 0.05). There were some disparities in therapist engagement (*P* < 0.05); however, this is more likely to reflect the implementation characteristics of different intervention methods (e.g., PPT vs. DBT) rather than baseline imbalance across studies. Given that the spread of core demographic characteristics was similar across study sites, the results continue to support the transferability hypothesis.

**Table 2:** Baseline comparability.

Variable	CBT (*N* = 4)	DBT (*N* = 2)	EBA (*N* = 3)	FTE (*N* = 4)	IR (*N* = 7)	MBE (*N* = 4)	PPT (*N* = 6)	RPE (*N* = 3)	SE (*N* = 4)	Con (*N* = 14)	*P*
Mean age	47.9 ± 3.3	45.5 ± 0.8	44.7 ± 0.8	45.7 ± 4.7	41.6 ± 5.3	40.8 ± 7.6	41.5 ± 4.5	46.3 ± 7.3	44.7 ± 4.1	45.2 ± 5.1	0.43
Baseline pain levels	4.9 ± 0.2	5 ± 0.7	6.7 ± 1.1	6.1 ± 1.7	5.5 ± 1.2	6 ± 1.8	5.8 ± 0.7	5.4 ± 1.2	4.5 ± 1.4	5.2 ± 1.3	0.35
intervention Duration (days)	44.2 ± 17.9	42 ± 19.8	45.7 ± 12.9	26.2 ± 18.4	39.9 ± 26.7	50.8 ± 39.3	30.3 ± 9.6	24 ± 8.7	50.5 ± 26.7	49.2 ± 26.7	0.70
Number of interventions	9.8 ± 3.5	8 ± 0	11.3 ± 4	11 ± 3.5	15.6 ± 7.7	32.5 ± 26	16 ± 7.9	22.3 ± 26.5	29.2 ± 23.5	13.2 ± 7.7	0.36
Intervention frequency (times/week)	2.8 ± 2.9	2.5 ± 0.7	2.2 ± 1.4	5.2 ± 5.9	3 ± 1.9	7.2 ± 4.8	3.3 ± 1.8	4.7 ± 3.2	7.1 ± 9.4	3.5 ± 5.5	0.60

#### 3.5.2 Pain

**Figure 4:**
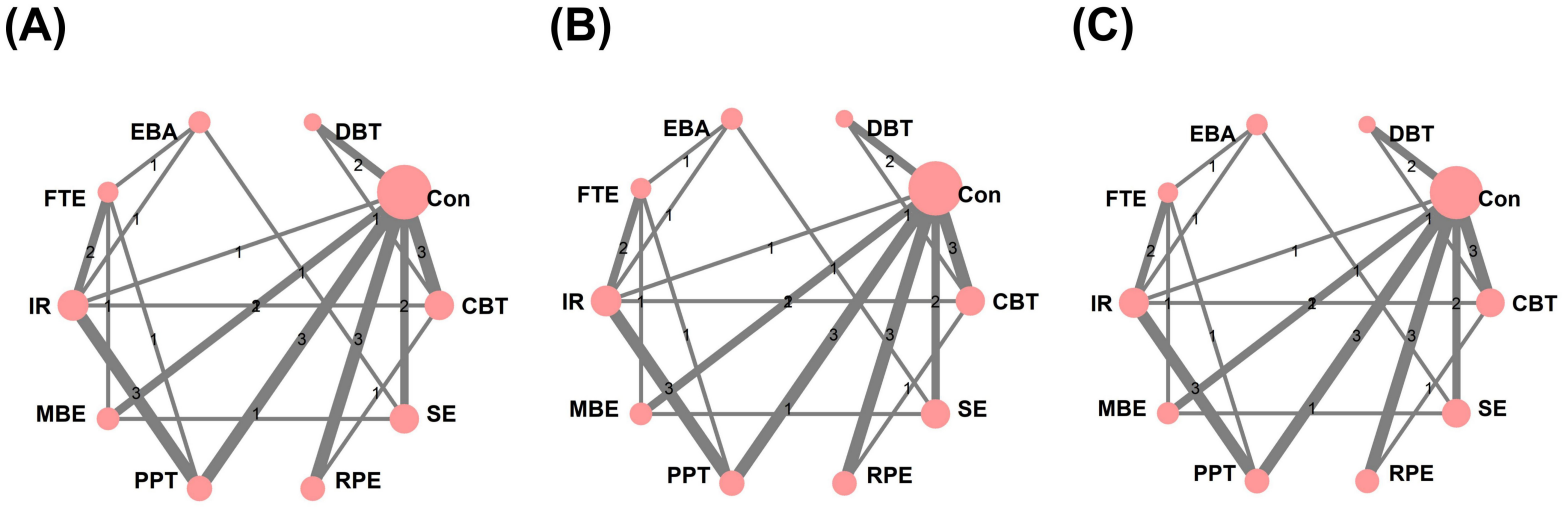
Network diagram of **(A)** Pain; **(B)** Depression; **(C)** Anxiety. CBT, Cognitive Behavioral Therapy; EBA, Education with Behavioral Activation; RPE, Relaxation and Psychological Education; MBE, Mind Body Exercise; SE, Structured Exercise; FTE, Functional and Targeted Exercise; PPT, Passive Physical Therapy; DBT, Digital and Biofeedback Therapy; IR, Integrated Rehabilitation.

**Figure 5:**
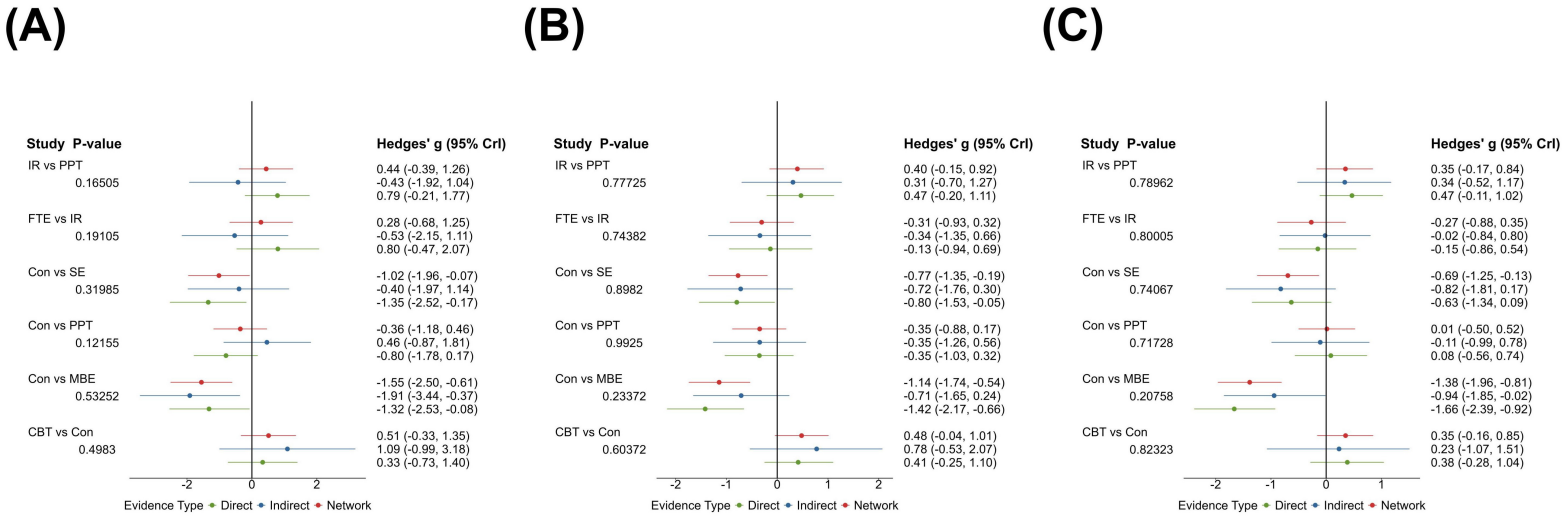
Local inconsistency of **(A)** Pain; **(B)** Depression; **(C)** Anxiety. Point colors indicate evidence source, green = direct evidence, blue = indirect evidence, red = network estimate. CBT, Cognitive Behavioral Therapy; EBA, Education with Behavioral Activation; RPE, Relaxation and Psychological Education; MBE, Mind Body Exercise; SE, Structured Exercise; FTE, Functional and Targeted Exercise; PPT, Passive Physical Therapy; DBT, Digital and Biofeedback Therapy; IR, Integrated Rehabilitation.

**Figure 6:**
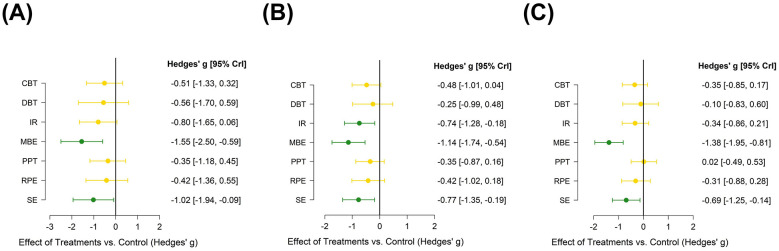
NMA forest plot of **(A)** Pain; **(B)** Depression; **(C)** Anxiety. Colors reflect the posterior probability of direction, green = beneficial (95 % CrI entirely below zero), gold = no clear difference (95 % CrI spans zero), red = harmful (95 % CrI entirely above zero). CBT, Cognitive Behavioral Therapy; EBA, Education with Behavioral Activation; RPE, Relaxation and Psychological Education; MBE, Mind Body Exercise; SE, Structured Exercise; FTE, Functional and Targeted Exercise; PPT, Passive Physical Therapy; DBT, Digital and Biofeedback Therapy; IR, Integrated Rehabilitation.

**Figure 7:**
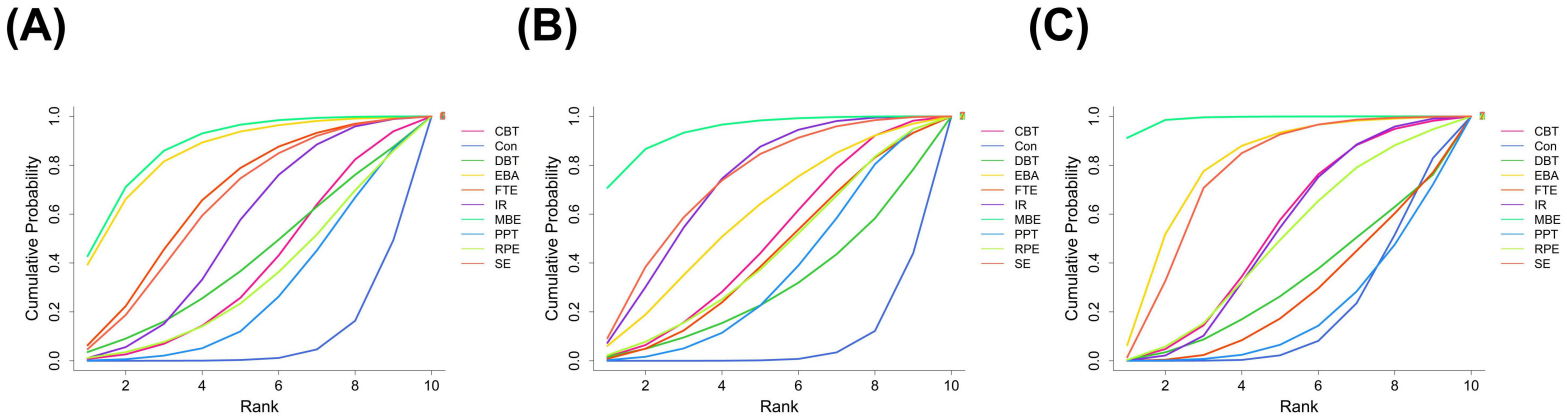
SUCRA probability ranking analysis of **(A)** Pain; **(B)** Depression; **(C)** Anxiety. The area under the curve represents the ranking of the corresponding intervention measures. CBT, Cognitive Behavioral Therapy; EBA, Education with Behavioral Activation; RPE, Relaxation and Psychological Education; MBE, Mind Body Exercise; SE, Structured Exercise; FTE, Functional and Targeted Exercise; PPT, Passive Physical Therapy; DBT, Digital and Biofeedback Therapy; IR, Integrated Rehabilitation.

The network plot for pain outcomes (Figure 4A) shows multiple closed loops within the evidence structure. And node splitting analysis reveals no meaningful inconsistencies between direct and indirect evidence for treatment contrasts (*p* > 0.05) (Figure 5A). The NMA forest plot (Figure 6A) demonstrated that, among the various non-pharmacological interventions, MBE (SMD: −1.55, 95% CrI [−2.50, −0.59]) and SE (SMD: −1.02, 95% CrI [−1.94, −0.09]) yielded a demonstrable lowering in pain relative to the control group. This finding was consistent with the ranking estimates presented in Supplementary Table S4 (SMD: −1.55, 95% CrI [−2.49, −0.59]; SMD: −1.01, 95% CrI [−1.96, −0.069]). SUCRA rankings (Figure 7A; Supplementary Figure S5A) further clarified the comparative performance of the interventions: MBE (87.5%)> EBA (84.8%)> FTE (66.2%)> SE (63.2%)> IR (52.5%)> control intervention (8%). Given the high degree of heterogeneity observed, these rankings should be interpreted with caution and are intended for reference only, rather than as definitive.

#### 3.5.3 Depression

The network diagram (Figure 4B) illustrates several closed loops. Node splitting analysis reveals that all comparison *p*-values are non-significant (*p* > 0.05) (Figure 5B). The NMA forest plot (Figure 6B) reveals that MBE [SMD: −1.14, 95% CrI (−1.74, −0.54)], IR [SMD: −0.74, 95% CrI (−1.28, −0.18)] and SE [SMD: −0.77, 95% CrI (−1.35, −0.19)] exhibited superiority compared to control interventions in mitigating depression. The league table (Supplementary Table S5 in the Supplementary Materials) similarly indicated that MBE [SMD: −1.14, 95% CrI (−1.72, −0.56)], IR [SMD: −0.74, 95% CrI (−1.29, −0.19)] and SE [SMD: −0.77, 95% CrI (−1.34, −0.19)] were substantially demonstrated greater efficacy than the control group. The SUCRA probability ranking (Figure 7B, Supplementary Figure S5B) produced the subsequent treatment hierarchy: MBE (93.9%)> SE (72.3%)> IR (71.8%)> EBA (58.3%)> control intervention (6.7%). Due to moderate heterogeneity, the ranking may not reflect a stable order of intervention.

#### 3.5.4 Anxiety

In the anxiety outcomes network (Figure 4C), node split tests indicated no significant discrepancies (all *p* > 0.05) (Figure 5C). The NMA forest plot (Figure 6C) indicated that both MBE [SMD: −1.38, 95% CrI (−1.95, −0.81)] and SE [SMD:−0.69, 95% CrI [−1.25, −0.14)] surpassed the control intervention. The league table (Supplementary Table S6 in the Supplementary Materials) produced the same outcomes. The SUCRA probability ranking (Figure 7C, Supplementary Figure S5C) produced the subsequent treatment hierarchy: MBE (98.8%)> EBA (79%)> SE (75.2%)> CBT (52.1%) > IR (50.9%) > control intervention (18.8%). Caution is warranted in interpreting these results.

### 3.6 Publication bias

**Figure 8:**
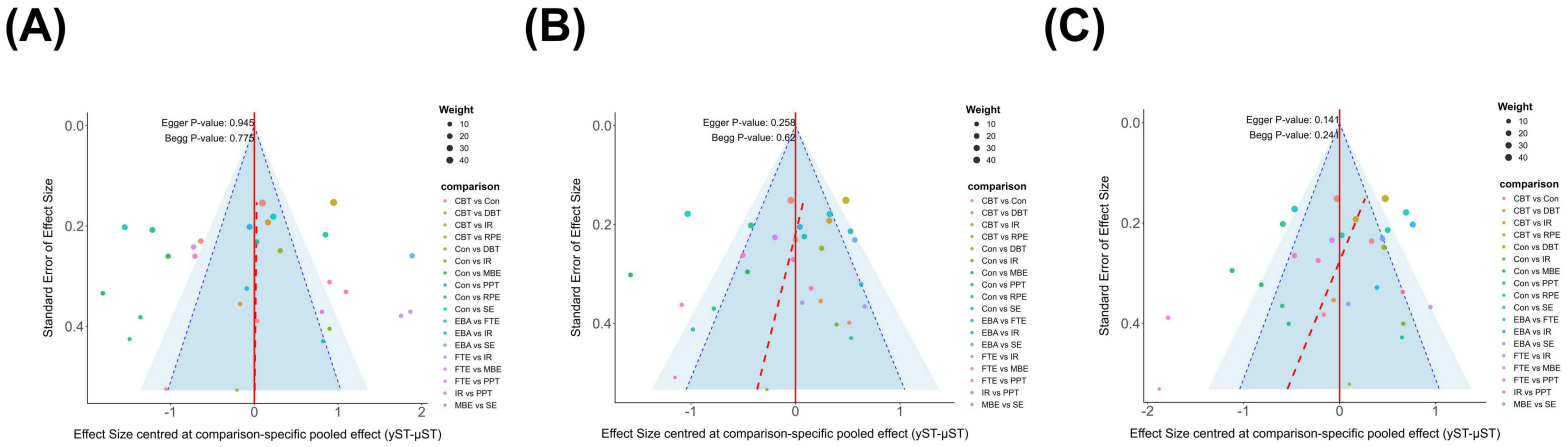
Funnel plot of **(A)** Pain; **(B)** Depression; **(C)** Anxiety. CBT, Cognitive Behavioral Therapy; EBA, Education with Behavioral Activation; RPE, Relaxation and Psychological Education; MBE, Mind Body Exercise; SE, Structured Exercise; FTE, Functional and Targeted Exercise; PPT, Passive Physical Therapy; DBT, Digital and Biofeedback Therapy; IR, Integrated Rehabilitation.

Funnel plots were examined for pain, depression, and anxiety outcomes in addition to Egger regression tests and Begg's rank correlation tests (Figure 8). No clear reporting bias was observed in the results. However, most of the included studies involved a small number of paired comparisons (Supplementary Table S7), and the relevant statistical tests may have struggled to detect small-sample effects; therefore, Interpretation of the results should be cautious. To further investigate the potential influence of selective reporting, a trim-and-fill analysis was performed. The assessment indicated that the impact on the estimates of the primary effects was minimal, suggesting that the study conclusions are robust to a certain extent. However, given the complex composition of the evidence network and the limited number of included studies, potential publication bias cannot be entirely ruled out.

### 3.7 Sensitivity analyses

Outliers were identified using box plots and Bayesian leverage plots (Supplementary Figures S6–S8), and a stepwise exclusion method was employed to quantify the impact of individual studies on the efficacy of interventions for pain, depression and anxiety. The results showed that the MBE provided robust improvements across all three outcomes, with consistently high SUCRA rankings in sensitivity analyses (pain 84%−90%, depression 80%−96%, anxiety 97%−99%); the statistical significance of the intervention effects remained unaffected (Supplementary Figures S9–S11 and Supplementary Tables S8–S10). IR performed reliably for the depression outcome, with the effect size remaining significant following stepwise exclusion (Supplementary Figure S10 and Supplementary Table S9). The effect of SE, however, was unstable; following the exclusion of Wang AS's study (30), its statistical significance for all outcomes disappeared (Supplementary Figures S9–S11 and Supplementary Tables S8–S10). Additional recalculations using fixed-effects models further confirmed the SUCRA rankings and statistical significance of MBE and SE, and the efficacy of IR for depression was validated (Supplementary Figures S12). However, as the models differ in their assumptions regarding heterogeneity, care should be taken in interpreting these results in light of the model assumptions.

### 3.8 Subgroup analyses

This study conducted a Bayesian subgroup interaction analysis on study quality, age, total number of interventions, baseline pain severity, duration of intervention, frequency of intervention, and mode of intervention (Supplementary Table S11) to explore potential moderating factors. The results showed that the 95% CrI for the mean difference across subgroups spanned zero, with low posterior probabilities of practical significance (0.64–0.89); no clear moderating factors were identified. The low-dose subgroup showed a certain trend in depression outcomes (Prob. of Positive = 0.979), but the posterior probability of interaction with the high-dose subgroup was 0.776, indicating considerable uncertainty. The effect estimates for patient-led intervention models were higher than those for therapist-led models in pain and depression outcomes (Prob. of Meaningful Diff. of 0.861 and 0.824, respectively), suggesting they may have clinical potential. However, the evidence for the aforementioned subgroup interactions was limited; overall, the intervention effects were consistent across subgroups.

### 3.9 Meta regression analyses

**Figure 9:**
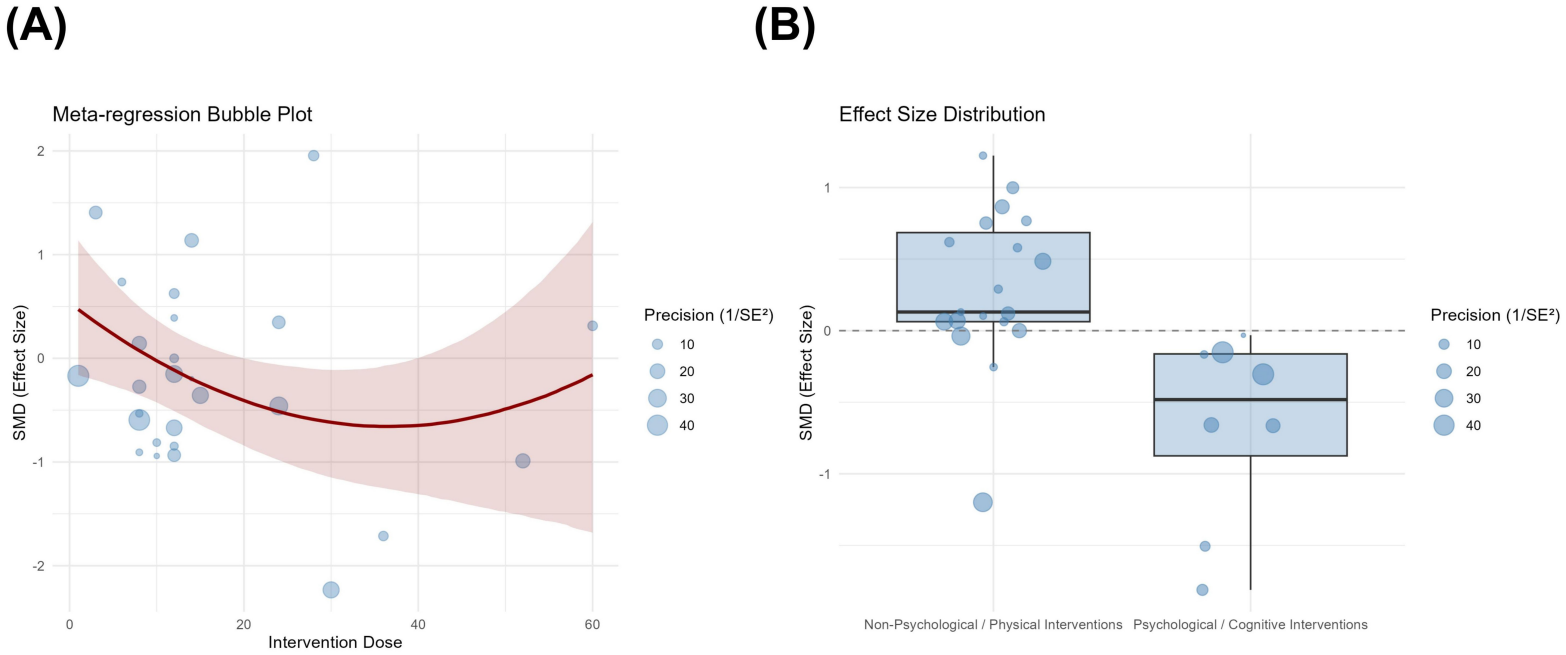
Bayesian meta-regression analysis **(A)** Dose-response curve for pain relief; **(B)** Distribution Chart of Anxiety Intervention Types. In **Figure A**, the bubbles represent the original studies, with bubble size proportional to the study weight (1/SE^2^). The solid red line represents the posterior median prediction curve from the Bayesian quadratic polynomial model, and the red shaded area represents the 95% credibility interval. In **Figure B**, the median line, the edges of the box and the whiskers represent the median effect size, the interquartile range and the full range, respectively.

Bayesian meta-regression analysis revealed that, for all three outcomes, most covariates did not meaningfully influence the effect of the intervention, with the 95% CrI for all including zero (Supplementary Tables S12–S14). The quadratic term for the total number of pain interventions was significant [β = −0.066, 95% CrI (−0.124, −0.009)], with the best fit (ΔLOO = −19.9), explaining 12.6% of the heterogeneity. The dose-response curve (Figure 9A) shows a U-shaped trend in pain relief, with efficacy first increasing and then decreasing as the number of interventions increased, peaking at approximately 30–40 sessions. Intervention type (psychological/cognitive vs. non-psychological/non-cognitive) was a significant moderator for anxiety outcomes [β = −0.469, 95% CrI (−0.871, −0.049)], accounting for 35.2% of the heterogeneity. The categorical meta-regression plot (Figure 9B) shows that the effect size for psychological/cognitive interventions lies below the zero line, with a lower median than that for non-psychological/physical interventions, suggesting that their analgesic effects may be more pronounced. No significant moderators were identified for the depression outcome. Residual heterogeneity remained high across all models (*I*^2^ = 80.6%−93.2%), indicating that the existing covariates cannot account for most of the variation between studies.

## 4 Discussion

### 4.1 Comparative effectiveness of different interventions

This study examined the relative efficacy of multiple non-drug interventions for patients with CLBP accompanied by emotional symptoms using a Bayesian NMA framework. The available evidence suggests that MBE may be effective in alleviating pain, anxiety and depression (moderate-quality evidence); however, the 95% CrI is wide, and as direct comparative studies are limited, there is some uncertainty regarding the effect estimates. SE showed a trend toward improvement for three outcomes (pain: low-quality evidence; anxiety and depression: moderate-quality evidence); however, sensitivity analyses indicated that the effects were highly dependent on individual studies, and findings should be viewed cautiously. IR indicates a putative advantage for depressive symptoms (moderate-quality evidence); however, the network connectivity is sparse, and robustness requires further validation. Although this study expands the assessment of interventions for pain and emotional comorbidity, due to high outcome heterogeneity, the SUCRA ranking is for reference only, and clinical decisions should not be based solely on the ranking results. Larger, high-quality RCTs remain necessary to confirm these findings.

### 4.2 Specific therapeutic efficacy and mechanism of action of intervention measures

In the management of chronic pain, exercise therapy not only alleviates pain by improving physical function and enhancing neuromuscular control through SE, but may also exert effects by modulating neurotransmitters and neurotrophic factors in the central nervous system. Studies have shown that regular exercise increases blood levels of brain-derived neurotrophic factor and regulates urea metabolic pathways, which is associated with its antidepressant effects (52). A systematic review by Hayden et al. supports the efficacy of exercise therapy in CLBP (53), and clinical guidelines identify exercise as one of the core management strategies (54); however, its effects are typically characterized by considerable heterogeneity (53). Sensitivity analyses in this study revealed that the robustness of SE is limited; after excluding the study by Wang AS et al. (30), the effect was no longer significant. This may be related to the high complexity of exercise interventions themselves, which are influenced by factors such as intervention type, exercise dose, level of supervision and patient adherence, leading to substantial variations in results across studies. Therefore, although the SE has a certain theoretical basis, its actual efficacy remains uncertain. In clinical practice, it should be used with caution, taking into account the patient's specific circumstances, and further RCTs should be conducted to standardize exercise prescriptions and clarify dosage parameters in order to verify its true efficacy in alleviating pain and comorbid emotional symptoms.

The efficacy of exercise interventions may vary depending on the exercise modality. A study by Hayden et al. (55) demonstrated that different types of exercise have a significant impact on pain and functional outcomes, suggesting that the choice of exercise modality may be a key factor influencing the effectiveness of the intervention. This study found that MBE demonstrated certain pain-related benefits and alleviated depression and anxiety. Because MBE requires practitioners to combine breathing with slow and regular movements, which helps stabilize mood and divert attention during exercise. Research also confirms that MBE can reduce stress response by enhancing self-awareness and can also suppress pain through the central analgesic pathway (56, 57). At the same time, the research of Gothe NP (58) and Fan C et al. (59) supported these findings. Investigations demonstrate that MBE, such as yoga and meditation, alleviate pain and emotional disturbances by inhibiting activity in emotion-related brain regions-including the amygdala and medial prefrontal cortex, while activating areas such as the insula, anterior cingulate cortex, and orbitofrontal cortex (58, 59). MBE can help people refocus their attention on more stable, non-threatening physical sensations by lowering sympathetic nervous system activity, according to an additional clinical study (60). These systems have cross-symptom effects, affecting the fundamental bio-psychological circuits that control anxiety and pain at the same time. This helps to explain some of the multiple advantages found in this study. The effectiveness of MBE in alleviating pain, increasing functional ability, and improving quality of life has been repeatedly supported by numerous earlier meta-analyses (61–63). In theory, individuals with chronic pain who also have emotional comorbidity would benefit more from IR (64), a multimodal rehabilitation approach that integrates muscular function, activity limits, and psychological suffering. Additionally, its all-encompassing mode of action has psychological and biological underpinnings for reducing depression (65–67). The findings of this study partially support this hypothesis, though the pain relief achieved through IR did not reach statistical significance. IR usually combines a variety of rehabilitation treatments (e.g., manual therapy, exercise training, relaxation techniques, etc.) that are more likely to enhance pain-related secondary outcomes, such as functional capacity, self-efficacy, or self-management skills (67). Improvements in depression are more closely associated with these characteristics. Higher dosages, longer treatment cycles, or therapies focusing on more particular analgesic mechanisms may be necessary for pain itself. As a result, IR shows lower direct effects on pain but potential benefits on emotional recovery.

### 4.3 Subgroup and meta-regression analyses: dose-response relationships and intervention models

Bayesian meta-regression analysis revealed that most covariates did not substantially affect the intervention outcomes; however, the total number of interventions showed a non-linear relationship with pain outcomes, whilst the type of intervention may have moderated anxiety outcomes. Pain relief exhibited a *U*-shaped effect pattern for the number of interventions, peaking at approximately 30–40 sessions before subsequently declining. Moderate-intensity interventions may help establish appropriate cognitive patterns, enhance self-management skills and induce neuromuscular adaptive changes (68); the decline in efficacy following the peak may be related to reduced adherence due to increased treatment burden or to patients' increased reliance on medical care, which undermines the psychological motivation for active rehabilitation (69), although the mechanisms require further validation. With regard to anxiety outcomes, interventions incorporating psychological or cognitive components were more effective than purely physical interventions, consistent with theories regarding the central mechanisms of chronic pain. Emotions such as anxiety are often associated with catastrophic thinking and fear-based behaviors (70), whilst cognitive behavioral therapy or mindfulness interventions can indirectly improve emotional and pain experiences by modulating psychological processes. Furthermore, subgroup analyses indicate that patient-led self-management models outperform therapist-led models in terms of pain and depression outcomes, suggesting that enhancing patient autonomy and self-efficacy may be key factors in the long-term benefits of non-pharmacological interventions. Low-dose interventions show a certain positive trend in depression outcomes, indirectly corroborating the results of dose-response analyses; however, the evidence remains subject to considerable uncertainty.

Despite the above analysis, the explained heterogeneity remains low. The complexity of non-pharmacological intervention classifications, the uneven distribution of covariates, the sparse network structure, and the restricted sample size may have constrained the detection of moderating effects. The current evidence is insufficient to provide intervention recommendations based on subgroup characteristics; consequently, the results are exploratory in nature. In clinical practice, optimizing the frequency of interventions and selecting intervention types based on comorbidity profiles may help improve clinical outcomes. However, in order to achieve personalized and precise interventions, future research should not only utilize large sample sizes and standardized dose reporting, but also employ network dose-response meta-analyses to overcome the limitations of categorical dose grouping and better describe the complex relationship between intervention intensity and treatment outcomes (71). This method is capable of simultaneously integrating dose information from different interventions. By constructing continuous dose-response curves, it is possible to estimate the potential optimal range of intervention for various non-pharmacological treatments of CLBP accompanied by emotional symptoms, thereby providing more valuable evidence to inform clinical decision-making.

## 5 Limitations

Although this study systematically evaluated non-pharmacological interventions for CLBP using a Bayesian NMA, several limitations remain. Firstly, the inclusion of only trials that reported pain, depression and anxiety simultaneously—primarily targeting patients with complex psychological characteristics—may give rise to selection bias, limiting the generalisability of the results to the broader CLBP population. Secondly, the large SMDs observed for some interventions may be influenced by network sparsity and small-sample bias, increasing the uncertainty of certain results; consequently, the SUCRA ranking results should not be regarded as conclusive clinical evidence. Thirdly, the assessment of publication bias in the NMA analysis is subject to methodological limitations. As most comparisons included few studies, the statistical power of funnel plots and Egger and Begg tests is limited; furthermore, as NMA applications require additional assumptions, their robustness is lower than that of traditional pairwise analyses. Finally, there is some overlap between intervention types, with considerable clinical heterogeneity; furthermore, inconsistencies in intervention design, total treatment duration, dosage and follow-up reporting hinder the quantification of dose-response relationships. In summary, the evidence provided by this study is preliminary. Future research could consider constructing independent networks for each outcome to expand the pool of included studies, reduce selection bias, and further explore personalized intervention strategies for patients with different comorbidity profiles.

## 6 Conclusions

Based on a Bayesian NMA, MBE and SE may help attenuate pain and emotional symptoms in patients with CLBP, and IR may also offer potential benefits for pain-related depression. However, due to high heterogeneity and uncertainty in effect estimates, these outcomes should be treated with caution, and the SUCRA ranking is for reference only. The findings are primarily applicable to patients with CLBP accompanied by mood disorders and are limited by short- to medium-term follow-up. Well-designed RCTs are still required subsequently, employing standardized outcome measures and clearly defined intervention parameters, to validate the rankings and relative efficacy reported in this study.

## Data Availability

Publicly available datasets were analyzed in this study. This data can be found here: all data used in this meta-analysis were retrieved from publicly available bibliographic databases, including PubMed, Embase, Cochrane Library, Web of Science, CINAHL, Scopus, CNKI, and Wanfang. The detailed search strategies and included studies are provided in the Supplementary material.
