# Supplementary material for: Comparative effectiveness of non-pharmacological interventions for depression and anxiety in chronic low back pain: a Bayesian network meta-analysis of randomized controlled trials
Source: Front Public Health. 2026 Apr 20;14:1765762. doi: 10.3389/fpubh.2026.1765762 (PMC13137130; doi:10.3389/fpubh.2026.1765762)
Supplement: Supplementary file 1 [file Table_1.docx]

**Supplementary Materials**

**Table S1** Search strategy

| Database | Search Number | Query |
| --- | --- | --- |
| 1. PubMed | 1 | ((low back pain[MeSH Terms]) OR (low back pain[Title/Abstract]) OR (lumbar pain[Title/Abstract])) |
|  | 2 | ((non-pharmacological) OR (non-drug) OR (acupuncture) OR (massage) OR (tuina) OR (manual therapy) OR (tai chi) OR (yoga) OR (qigong) OR (exercise) OR (physical therapy) OR (cognitive behavioral therapy) OR (CBT)) |
|  | 3 | ((anxiety[MeSH Terms]) OR (depression[MeSH Terms]) OR (anxiety[Title/Abstract]) OR (depression[Title/Abstract]) OR (HAMA) OR (HAMD) OR (BDI) OR (SAS) OR (SDS)) |
|  | 4 | (randomized controlled trial[Publication Type]) OR (randomized[Title/Abstract]) OR (RCT[Title/Abstract]) |
|  | 5 | (humans[MeSH Terms]) |
| 2. Embase | #1 | low back pain'/exp OR 'lumbar pain':ti,ab OR 'low back pain':ti,ab |
|  | #2 | non pharmacological therapy'/exp OR 'complementary therapy'/exp OR acupuncture:ti,ab OR massage:ti,ab OR tuina:ti,ab OR 'manual therapy':ti,ab OR 'tai chi':ti,ab OR yoga:ti,ab OR qigong:ti,ab OR exercise:ti,ab OR 'physical therapy':ti,ab OR physiotherapy:ti,ab OR rehabilitation:ti,ab OR 'cognitive behavioral therapy':ti,ab OR CBT:ti,ab OR mindfulness:ti,ab |
|  | #3 | anxiety/exp OR depression/exp OR anxiety:ti,ab OR depression:ti,ab OR HAMA:ti,ab OR HAMD:ti,ab OR BDI:ti,ab OR SAS:ti,ab OR SDS:ti,ab |
|  | #4 | randomized controlled trial'/exp OR randomized:ti,ab OR RCT:ti,ab |
|  | #5 | 1 AND 2 AND 3 AND 4 |
| 3. Cochrane Library | #1 | (low back pain OR lumbar pain):ti,ab,kw |
|  | #2 | (non-pharmacological OR non-drug OR acupuncture OR massage OR tuina OR manual therapy OR tai chi OR yoga OR qigong OR exercise OR physical therapy OR physiotherapy OR rehabilitation OR cognitive behavioral therapy OR CBT OR mindfulness):ti,ab,kw |
|  | #3 | (anxiety OR depression OR HAMA OR HAMD OR BDI OR SAS OR SDS):ti,ab,kw |
|  | #4 | (randomized controlled trial OR RCT OR randomized):ti,ab,kw |
|  | #5 | #1 AND #2 AND #3 AND #4 |
| 4. Scopus | #1 | TITLE-ABS-KEY("low back pain" OR "lumbar pain") |
|  | #2 | TITLE-ABS-KEY("non-pharmacological" OR "non-drug" OR "acupuncture" OR "massage" OR "tuina" OR "manual therapy" OR "tai chi" OR "yoga" OR "qigong" OR "exercise" OR "physical therapy" OR "cognitive behavioral therapy" OR "CBT") |
|  | #3 | TITLE-ABS-KEY("anxiety" OR "depression" OR "HAMA" OR "HAMD" OR "BDI" OR "SAS" OR "SDS") |
|  | #4 | (TITLE-ABS-KEY("randomized controlled trial" OR "RCT" OR "randomized")) |
|  | #5 | TITLE-ABS-KEY("randomized controlled trial" OR "RCT" OR "randomized") |
|  | #6 | LIMIT-TO(DOCTYPE, "ar") |
|  | #7 | LIMIT-TO(SUBJAREA, "MEDI") OR LIMIT-TO(SUBJAREA, "NURS") OR LIMIT-TO(SUBJAREA, "HEAL") |
| 5. Cinahl | S1 | (MH "Low Back Pain") OR "low back pain" OR "lumbar pain" |
|  | S2 | (MH "Complementary Therapies+") OR "non-pharmacological" OR "non-drug" OR acupuncture OR massage OR tuina OR "manual therapy" OR "tai chi" OR yoga OR qigong OR exercise OR "physical therapy" OR physiotherapy OR rehabilitation OR "cognitive behavioral therapy" OR CBT OR mindfulness |
|  | S3 | (MH "Anxiety+") OR (MH "Depression+") OR anxiety OR depression OR HAMA OR HAMD OR BDI OR SAS OR SDS |
|  | S4 | (MH "Randomized Controlled Trials") OR randomized OR RCT |
|  | S5 | S1 AND S2 AND S3 AND S4 |
| 6. Web of Science | 1 | TS = ((low back pain OR lumbar pain) |
|  | 2 | TS = (non-pharmacological OR non-drug OR acupuncture OR massage OR tuina OR manual therapy OR tai chi OR yoga OR qigong OR exercise OR physical therapy OR physiotherapy OR rehabilitation OR cognitive behavioral therapy OR CBT OR mindfulness) |
|  | 3 | TS =(anxiety OR depression OR HAMA OR HAMD OR BDI OR SAS OR SDS) |
|  | 4 | TS =(randomized OR "randomized controlled trial" OR RCT) |

|  |
| --- |

**Table S2** Author, year of publication, sample size used in study and control groups, Mean age, sex, intervention duration, and outcome measures

| Study  （ID） | Year | Country | Sample size (n) | Gender (M/F) | Mean age | intervention duration | Outcome |
| --- | --- | --- | --- | --- | --- | --- | --- |
| Darnall BD  (1) | 2021 | USA | RPE: 87;  CBT: 88;  Con: 88 | 131/132 | RPE: 49.7; CBT: 45.9; Con: 48 | 30d | NRS, PROMIS, PCS, PSEQ |
| Dayanlr IO  (2) | 2020 | Turkey | PPT: 16;  FTE: 16;  IR: 16 | 46/8 | PPT: 34.56; FTE: 39.25; IR: 32.62 | 42d | VAS, STAI, BDI, MTrP, PPT, AROM, ODI |
| Espin A  (3) | 2025 | Spain | SE: 49;  Con: 50 | 124/6 | SE: 49; Con: 50 | 84d | NRS, GADS, SHS, EQ-5D, SQS, 5STS, KPU, SITTET |
| Field T  (4) | 2007 | USA | PPT: 15;  Con: 15 | 14/16 | PPT: 41; Con: 41 | 35d | VAS, STAI, POMS-D, Sleep Scale, ROM |
| Gardner T  (5) | 2019 | Australia | EBA: 37;  SE: 38 | 43/32 | EBA: 44; SE: 45 | 60d | NRS, DASS, QBPDS, SF-36, TSK, PSEQ |
| Gevers-Montor-o C  (6) | 2024 | Canada and Spain | PPT: 49;  Con: 49 | 31/22 | PPT: 48.3; Con: 48.3 | 28d | NRS, GAD, BDI, ODI, PCS, FABQ, TSK-11, PGIC, PPTs |
| Hernandez-Rei-f M  (7) | 2001 | USA | PPT: 13;  Con: 12 | 13/11 | PPT: 43.8; Con: 36.7 | 35d | SF-MPQ, VITAS, STAI, SCL-90-R, POMS-D, Sleep Scale, ROM, Cortisol, Catecholamines, Serotonin |
| Lazaridou A  (8) | 2023 | USA | DBT: 37;  Con: 29 | Predominant-ly female | DBT: 46; Con: 43.5 | 56d | BPI, HADS, ODI, EMG, QST |
| Liu MS  (9) | 2023 | China | MBE: 25;  Con: 25 | 33/17 | MBE: 44.4;  Con: 47.96 | 84d | VAS, HAMA, HAMD, SF-36 |
| Newton-John TR  (10) | 1995 | UK | CBT: 16;  DBT: 16;  Con: 12 | 27/17 | CBT: 44.37; DBT: 44.93;  Con: 47.72 | 28d | pain diary, STAI, BDI, CSQ, PDI, PBQ, GALS |
| Petrozzi MJ  (11) | 2019 | Australia and New Zealand | CBT: 54;  IR: 54 | 54/54 | CBT: 50.1; IR: 50.6 | 56d | PNRS, DASS21, RMD, PSFS, WAI, PSEQ, PCS |
| Rim M  (12) | 2022 | France | EBA: 50;  IR: 50 | 24/76 | EBA: 45.6; IR: 42.9 | 35d | VAS, HADS, EIFEL, FABQ, TSK |
| Ruan LH  (13) | 2022 | China | RPE: 42;  Con: 42 | 54/30 | RPE: 37.96; Con: 38.67 | 28d | VAS, SAS, SDS, ODI, RMDQ |
| Schlicker S  (14) | 2020 | Germany | CBT: 40;  Con: 36 | 55/21 | CBT: 51.3; Con: 50.1 | 63d | Pain ratings, HADS, CES-D, QIDS-SR16, AQOL-6D, OSWESTRY, PSEQ, MDQ, SEP, CSQ |
| Tekur P  (15) | 2012 | India | MBE: 40;  FTE: 40 | 36/44 | MBE: 49; FTE: 48 | 7d | VAS, STAI, BDI, SAR |
| Thanyawinichk-ul K  (16) | 2022 | Thailand | RPE: 10;  Con: 12 | 16/6 | RPE: 51.3; Con: 53.5 | 14d | BPI, HADS, Overall change |
| Trapp W  (17) | 2014 | Germany | FTE: 15;  IR: 15 | 11/19 | FTE: 45.53; IR: 40.6 | 14d | VAS, PASS, BDI, HAMD, PVAQ, PCS, MPI |
| van Erp R M A  (18) | 2021 | Netherlands | IR: 12;  Con: 13 | 14/11 | IR: 43; Con: 45 | 90d | NRS, HADS, QBPDS, PCS, TSK, PSEQ, GPE |
| Vignesh C  (19) | 2024 | India | MBE: 32;  Con: 34 | 37/29 | MBE: 38.45; Con: 37.8 | 84d | NRS, DASS-21, PSQI |
| Wang AS  (20) | 2023 | China | SE: 80;  Con: 80 | 74/86 | SE: 45.69; Con: 44.63 | 30d | VAS, HADS, ODI, SF-36 |
| Wang SA  (21) | 2024 | China | IR: 39  PPT: 25 | 55/65 | IR: 40.25  PPT: 40.61 | 28d | VAS, SAS, SDS, ODI, RMDQ, JOA |
| Yamada AS  (22) | 2023 | Brazil | EBA: 20  FTE: 20 | 27/13 | EBA: 44.5  FTE: 50.1 | 42d | NPS, HADS, HAD, SCSI, RMDQ, PSC, TSK, SF-6D, PPT, TS, CPM, NDQ |
| Zheng F  (23) | 2022 | China | MBE: 19  SE: 18 | 28/9 | MBE: 31.5  SE: 39.2 | 28d | NRS, GAD-7, PHQ-9, PCS, PSEQ, RMDQ |
| Miao YD  (24) | 2025 | China | IR: 30  PPT: 30 | 35/25 | IR: 41.23  PPT: 40.57 | 14d | VAS, SAS, SDS, PSQI |

**Note:** M/F: Males/females; CBT: Cognitive Behavioral Therapy; EBA: Education with Behavioral Activation; RPE: Relaxation and Psychological Education; MBE: Mind Body Exercise; SE: Structured Exercise; FTE: Functional and Targeted Exercise; PPT: Passive Physical Therapy; DBT: Digital and Biofeedback Therapy; IR: Integrated Rehabilitation.

**Table S3** Outcome measures and data included in the study.

| Study  ID | Scale (Pain,  Depression, Anxiety) | Times | Group | Pain  Mean (SD) | Depression  Mean (SD) | Anxiety  Mean (SD) | Data type | Direction |
| --- | --- | --- | --- | --- | --- | --- | --- | --- |
| (1) | NRS, PROMIS | 30d | RPE / CBT / Con | -0.79 (1.81) / -1.56 (1.78) / -0.48 (1.84) | -2.4 (9.20) / -2.49 (8.21) / -0.48 (8.41) | -2.55 (9.91) / -3.66 (7.80) / -1.14 (8.64) | Change scores | ↑worse* |
| (2) | VAS, BDI, STAI | 42d | PPT / FTE / IR | -3 (1.67) /  -4.5 (1.78) /  -3 (1.67) | -7.06 (4.62) / -4.56 (3.46) / -6.18 (3.85) | -18.25 (5.03) / -11.31 (5.99) / -14.87 (6.27) | Change scores | ↑worse |
| (3) | NRS, GADS | 84d | SE / Con | -1 (2.31) /  0.1 (2.45) | -0.3 (1.91) / -0.1 (1.93) | -0.6 (2.55) / -0.5 (2.70) | Change scores | ↑worse |
| (4) | VAS, POMS-D, STAI | 35d | PPT / Con | -3.7 (2.52) / -1.7 (2.26) | -5.1 (11.20) / -0.9 (6.69) | -9.6 (11.78) / -10.3 (9.96) | Change scores | ↑worse |
| (5) | NRS, DASS | 60d | EBA / SE | -3.3 (1.71) / -0.9 (1.67) | -1.4 (8.36) / -0.2 (10.79) | -0.2 (7.35) / -0.2 (8.55) | Change scores | ↑worse |
| (6) | NRS, BDI, GAD | 28d | PPT / Con | -30.9 (15.94) / -17.9 (22.05) | -2.9 (7.37) / -2.3 (6.26) | -1.3 (4.57) / -1.6 (4.03) | Change scores | ↑worse |
| (7) | VITAS, POMS-D, STAI, | 35d | PPT / Con | -3.9 (2.25) / -1.6 (2.48) | -7.4 (11.97) / -0.6 (7.91) | -7.4 (10.45) / -8.8 (10.32) | Change scores | ↑worse |
| (8) | BPI, HADS | 56d | DBT / Con | -0.9 (1.64) / -0.4 (2.00) | -0.1 (3.42) /  0 (3.60) | 0.3 (4.06) /  -0.2 (4.20) | Change scores | ↑worse |
| (9) | VAS, HAMA, HAMD, | 84d | MBE / Con | -3.21 (0.64) / -1.97 (0.78) | -11.28 (2.44) / -8.92 (3.08) | -9.48 (2.34) / -5.32 (3.05) | Change scores | ↑worse |
| (10) | pain diary, BDI, STAI | 28d | CBT / DBT / Con | -5.34 (12.87) / -8.39 (10.10) / 1.19 (10.47) | -5.94 (8.35) / -4.13 (5.68) / 0.25 (6.22) | -2.07 (12.46) / -3 (12.00) / -0.17 (8.90) | Change scores | ↑worse |
| (11) | PNRS, DASS21 | 56d | IR / CBT | -2 (2.03) /  -2.3 (1.91) | -1.3 (4.95) / -1.1 (4.70) | -0.6 (3.46) / -0.4 (2.81) | Change scores | ↑worse |
| (12) | VAS, HADS | 35d | EBA / IR | -2.5 (2.13) / -3.2 (1.73) | -0.32 (2.61) / -1.8 (2.10) | -2 (4.32) /  -0.1 (3.40) | Change scores | ↑worse |
| (13) | VAS, SAS, SDS | 28d | RPE / Con | -4.01 (0.82) / -3.28 (0.63) | -8.47 (4.66) / -5.04 (5.43) | -10.25 (7.07) / -5.6 (6.92) | Change scores | ↑worse |
| (14) | Pain ratings, HADS, CES-D, | 63d | CBT / Con | 0 (1.90) /  -0.27 (1.84) | -6.84 (7.94) / -4.64 (7.22) | -2.84 (3.45) / -0.6 (3.21) | Change scores | ↑worse |
| (15) | VAS, BDI, STAI | 7d | MBE / FTE | -3.28 (1.85) / -1.03 (2.06) | -5.7 (8.33) / -2.6 (6.10) | -8.59 (9.06) / -0.52 (9.40) | Change scores | ↑worse |
| (16) | BPI, HADS | 14d | RPE / Con | -0.7 (1.49) / -0.33 (2.03) | -2 (3.30) / -1.33 (2.46) | -1.3 (3.69) / -1.17 (4.10) | Change scores | ↑worse |
| (17) | VAS, BDI, PASS | 14d | FTE / IR | -1.36 (1.82) / -0.04 (1.67) | -1.73 (5.69) / -0.87 (4.34) | -8.93 (39.86) / 1.4 (28.35) | Change scores | ↑worse |
| (18) | NRS, HADS | 90d | IR / Con | 0.2 (2.62) /  -0.8 (2.35) | -1 (3.83) /  0 (3.71) | -0.6 (4.02) /  -1 (3.58) | Change scores | ↑worse |
| (19) | NRS, DASS-21 | 84d | MBE / Con | -3.56 (3.13) / -0.58 (3.17) | -5.36 (3.13) / 0.84 (3.14) | -8.73 (4.95) / 1.47 (6.09) | Change scores | ↑worse |
| (20) | VAS, HADS | 30d | SE / Con | -2.7 (0.54) / -1.5 (0.53) | -6.3 (2.49) / -2.58 (2.57) | -6.09 (2.92) / -2.34 (3.30) | Change scores | ↑worse |
| (21) | VAS, SAS, SDS | 28d | IR / PPT | -3.98 (1.13) / -1.72 (1.16) | -11.16 (6.03) / -5.44 (5.53) | -7.27 (5.96) / -2.66 (6.21) | Change scores | ↑worse |
| (22) | NPS, HADS | 42d | EBA / FTE | -4.7 (2.37) / -3.15 (2.49) | -2.15 (3.97) / -0.45 (3.20) | -4.25 (3.39) / -1.55 (3.51) | Change scores | ↑worse |
| (23) | NRS, GAD-7, PHQ-9 | 28d | MBE / SE | -1.237 (1.53) / -0.747 (1.53) | -1.092 (3.03) / -0.792 (3.11) | -1.215 (2.68) / 0.498 (2.74) | Change scores | ↑worse |
| (24) | VAS, SAS, SDS | 14d | IR / PPT | -4.25 (0.74) / -4.06 (0.19) | -13.21 (10.36) / -7.92 (11.13) | -11.36 (3.74) / -7.04 (4.74) | Change scores | ↑worse |

**Note:** ^*^ The higher the score, the more severe the symptoms.

NRS: Numeric Rating Scale; PNRS: Pictorial Numeric Rating Scale; NPS: Numeric Pain Scale; VAS: Visual Analogue Scale; BPI: Brief Pain Inventory; Pain diary: Pain Diary; Pain ratings: Pain Rating Scale; VITAS: Visual Intensive Tabular Anaesthesia Scale; PROMIS: Patient-Reported Outcomes Measurement Information System; BDI: Beck Depression Inventory; CES-D: Center for Epidemiologic Studies Depression Scale; HAMD: Hamilton Depression Rating Scale; PHQ-9: Patient Health Questionnaire-9; SDS: Self-rating Depression Scale; POMS-D: Profile of Mood States–Depression Subscale; STAI: State-Trait Anxiety Inventory; HADS: Hospital Anxiety and Depression Scale; GAD-7: Generalized Anxiety Disorder-7; HAMA: Hamilton Anxiety Rating Scale; SAS: Self-rating Anxiety Scale; PASS: Pain Anxiety Symptoms Scale; DASS: Depression Anxiety Stress Scales; DASS-21: 21-item Depression Anxiety Stress Scales; GADS: Goldberg Anxiety and Depression Scale; CBT: Cognitive Behavioral Therapy; EBA: Education with Behavioral Activation; RPE: Relaxation and Psychological Education; MBE: Mind Body Exercise; SE: Structured Exercise; FTE: Functional and Targeted Exercise; PPT: Passive Physical Therapy; DBT: Digital and Biofeedback Therapy; IR: Integrated Rehabilitation.

**Table S4** League Table of pain

| Con |  |  |  |  |  |  |  |  |  |
| --- | --- | --- | --- | --- | --- | --- | --- | --- | --- |
| -0.8 (-1.66, 0.07) | IR |  |  |  |  |  |  |  |  |
| -0.51 (-1.35, 0.34) | 0.29 (-0.75, 1.33) | CBT |  |  |  |  |  |  |  |
| -0.57 (-1.71, 0.57) | 0.23 (-1.17, 1.62) | -0.06 (-1.36, 1.2) | DBT |  |  |  |  |  |  |
| -1.55 (-2.49, -0.59) | -0.75 (-1.9, 0.38) | -1.04 (-2.28, 0.18) | -0.98 (-2.45, 0.49) | MBE |  |  |  |  |  |
| -0.36 (-1.17, 0.46) | 0.44 (-0.37, 1.25) | 0.15 (-0.96, 1.23) | 0.21 (-1.18, 1.59) | 1.19 (0.03, 2.36) | PPT |  |  |  |  |
| -0.41 (-1.35, 0.55) | 0.38 (-0.87, 1.63) | 0.09 (-1.05, 1.24) | 0.16 (-1.28, 1.62) | 1.13 (-0.21, 2.46) | -0.05 (-1.27, 1.19) | RPE |  |  |  |
| -1.01 (-1.96, -0.06) | -0.21 (-1.38, 0.94) | -0.5 (-1.75, 0.73) | -0.44 (-1.92, 1.01) | 0.54 (-0.57, 1.64) | -0.65 (-1.84, 0.52) | -0.59 (-1.93, 0.73) | SE |  |  |
| -1.51 (-2.72, -0.31) | -0.72 (-1.85, 0.4) | -1.01 (-2.41, 0.37) | -0.94 (-2.59, 0.69) | 0.04 (-1.33, 1.36) | -1.15 (-2.41, 0.11) | -1.1 (-2.61, 0.41) | -0.51 (-1.74, 0.7) | EBA |  |
| -1.08 (-2.15, -0.01) | -0.28 (-1.25, 0.67) | -0.57 (-1.84, 0.71) | -0.51 (-2.06, 1.04) | 0.47 (-0.67, 1.63) | -0.72 (-1.79, 0.35) | -0.67 (-2.08, 0.75) | -0.07 (-1.32, 1.21) | 0.43 (-0.74, 1.6) | FTE |

**Note:** CBT: Cognitive Behavioral Therapy; EBA: Education with Behavioral Activation; RPE: Relaxation and Psychological Education; MBE: Mind Body Exercise; SE: Structured Exercise; FTE: Functional and Targeted Exercise; PPT: Passive Physical Therapy; DBT: Digital and Biofeedback Therapy; IR: Integrated Rehabilitation.

**Table S5** League Table of Depression

| Con |  |  |  |  |  |  |  |  |  |
| --- | --- | --- | --- | --- | --- | --- | --- | --- | --- |
| -0.74 (-1.29, -0.19) | IR |  |  |  |  |  |  |  |  |
| -0.48 (-1.01, 0.06) | 0.26 (-0.39, 0.91) | CBT |  |  |  |  |  |  |  |
| -0.24 (-0.99, 0.5) | 0.5 (-0.4, 1.38) | 0.24 (-0.58, 1.07) | DBT |  |  |  |  |  |  |
| -1.14 (-1.72, -0.56) | -0.4 (-1.13, 0.31) | -0.66 (-1.44, 0.1) | -0.9 (-1.83, 0.04) | MBE |  |  |  |  |  |
| -0.35 (-0.88, 0.18) | 0.39 (-0.14, 0.91) | 0.13 (-0.56, 0.82) | -0.1 (-1.01, 0.8) | 0.8 (0.05, 1.53) | PPT |  |  |  |  |
| -0.43 (-1.02, 0.17) | 0.31 (-0.47, 1.1) | 0.05 (-0.66, 0.77) | -0.18 (-1.12, 0.75) | 0.72 (-0.12, 1.55) | -0.08 (-0.86, 0.71) | RPE |  |  |  |
| -0.77 (-1.34, -0.19) | -0.03 (-0.75, 0.67) | -0.29 (-1.05, 0.47) | -0.53 (-1.45, 0.41) | 0.37 (-0.32, 1.08) | -0.42 (-1.15, 0.32) | -0.34 (-1.17, 0.49) | SE |  |  |
| -0.62 (-1.37, 0.14) | 0.13 (-0.6, 0.83) | -0.14 (-1.02, 0.73) | -0.37 (-1.43, 0.68) | 0.53 (-0.33, 1.39) | -0.27 (-1.08, 0.54) | -0.19 (-1.15, 0.75) | 0.15 (-0.6, 0.92) | EBA |  |
| -0.43 (-1.12, 0.26) | 0.31 (-0.31, 0.93) | 0.04 (-0.76, 0.85) | -0.19 (-1.18, 0.82) | 0.71 (-0.02, 1.44) | -0.09 (-0.78, 0.63) | -0.01 (-0.92, 0.89) | 0.33 (-0.46, 1.12) | 0.18 (-0.58, 0.94) | FTE |

**Note:** CBT: Cognitive Behavioral Therapy; EBA: Education with Behavioral Activation; RPE: Relaxation and Psychological Education; MBE: Mind Body Exercise; SE: Structured Exercise; FTE: Functional and Targeted Exercise; PPT: Passive Physical Therapy; DBT: Digital and Biofeedback Therapy; IR: Integrated Rehabilitation.

**Table S6** League Table of Anxiety

| Con |  |  |  |  |  |  |  |  |  |
| --- | --- | --- | --- | --- | --- | --- | --- | --- | --- |
| -0.33 (-0.85, 0.21) | IR |  |  |  |  |  |  |  |  |
| -0.35 (-0.85, 0.15) | -0.02 (-0.65, 0.6) | CBT |  |  |  |  |  |  |  |
| -0.11 (-0.84, 0.62) | 0.22 (-0.67, 1.08) | 0.24 (-0.59, 1.05) | DBT |  |  |  |  |  |  |
| -1.38 (-1.96, -0.79) | -1.05 (-1.76, -0.36) | -1.03 (-1.78, -0.28) | -1.27 (-2.19, -0.34) | MBE |  |  |  |  |  |
| 0.02 (-0.49, 0.52) | 0.34 (-0.17, 0.85) | 0.37 (-0.31, 1.03) | 0.12 (-0.75, 1.01) | 1.4 (0.69, 2.11) | PPT |  |  |  |  |
| -0.31 (-0.88, 0.27) | 0.02 (-0.75, 0.78) | 0.04 (-0.63, 0.73) | -0.2 (-1.12, 0.72) | 1.07 (0.26, 1.9) | -0.32 (-1.09, 0.43) | RPE |  |  |  |
| -0.69 (-1.25, -0.13) | -0.36 (-1.07, 0.33) | -0.34 (-1.07, 0.39) | -0.58 (-1.51, 0.33) | 0.69 (0, 1.36) | -0.71 (-1.41, 0) | -0.38 (-1.18, 0.41) | SE |  |  |
| -0.77 (-1.49, -0.02) | -0.44 (-1.13, 0.23) | -0.42 (-1.25, 0.41) | -0.66 (-1.68, 0.37) | 0.61 (-0.21, 1.43) | -0.79 (-1.56, -0.02) | -0.46 (-1.39, 0.45) | -0.08 (-0.81, 0.66) | EBA |  |
| -0.06 (-0.72, 0.62) | 0.27 (-0.34, 0.9) | 0.3 (-0.49, 1.08) | 0.05 (-0.93, 1.04) | 1.33 (0.62, 2.04) | -0.07 (-0.75, 0.62) | 0.25 (-0.61, 1.13) | 0.64 (-0.12, 1.42) | 0.72 (0, 1.45) | FTE |

**Note:** CBT: Cognitive Behavioral Therapy; EBA: Education with Behavioral Activation; RPE: Relaxation and Psychological Education; MBE: Mind Body Exercise; SE: Structured Exercise; FTE: Functional and Targeted Exercise; PPT: Passive Physical Therapy; DBT: Digital and Biofeedback Therapy; IR: Integrated Rehabilitation.

**Table S7** Assessment of publication bias and the small-study effect

| Outcome | Total Studies (N) | Comparison Type | k (Trials) | Egger’s Test (p-value) | Begg’s Test (p-value) | Trim-and-Fill (Studies Filled) | Impact on Results | Sample Size (n) |
| --- | --- | --- | --- | --- | --- | --- | --- | --- |
| Pain | 24 | Global Network | 30* | 0.945 | 0.775 | / | Stable |  |
|  |  | CBT vs Con | 3 | 0.818 | 1 | 0 | No change | 280 |
|  |  | Con vs RPE | 3 | 0.573 | 1 | 0 | No change | 281 |
|  |  | IR vs PPT | 3 | 0.725 | 1 | 0 | No change | 156 |
|  |  | Con vs PPT | 3 | 0.051 | 0.333 | 2 | Stable | 153 |
|  |  | Other 14 types | 1–2 | / | / | / | Limited Power | / |
| Depression | 24 | Global Network | 30* | 0.258 | 0.62 | -- | Stable |  |
|  |  | CBT vs Con | 3 | 0.283 | 0.333 | 0 | No change | 280 |
|  |  | Con vs RPE | 3 | 0.498 | 1 | 0 | No change | 281 |
|  |  | IR vs PPT | 3 | 0.248 | 1 | 1 | Stable | 156 |
|  |  | Con vs PPT | 3 | 0.086 | 0.333 | 2 | Stable | 153 |
|  |  | Other 14 types | 1–2 | / | / | / | Limited Power | / |
| Anxiety | 24 | Global Network | 30* | 0.142 | 0.241 | / | Stable |  |
|  |  | CBT vs Con | 3 | 0.951 | 1 | 0 | No change | 280 |
|  |  | Con vs RPE | 3 | 0.426 | 1 | 1 | Stable | 281 |
|  |  | IR vs PPT | 3 | 0.1 | 1 | 0 | No change | 156 |
|  |  | Con vs PPT | 3 | 0.103 | 1 | 0 | No change | 153 |
|  |  | Other 14 types | 1–2 | / | / | / | Limited Power | / |

**Note:** Stable: indicates that the direction and significance of the pooled effect size remain unchanged after including studies with missing data in the analysis. /: Due to insufficient degrees of freedom, no statistical tests were performed for comparisons where n < 3. CBT: Cognitive Behavioral Therapy; RPE: Relaxation and Psychological Education; PPT: Passive Physical Therapy; IR: Integrated Rehabilitation.

**Table S8** Leave-One-Out sensitivity analysis results of Pain

| Treat-ment | Overall Baseline | Removed Study | | | | | | | |
| --- | --- | --- | --- | --- | --- | --- | --- | --- | --- |
|  |  | Study14 | Study24 | Study9 | Study3 | Study1 | Study20 | Study21 | Study13 |
| SUCRA RANK (%) | | | | | | | | | |
| MBE | 87.70 | 86.29 | 87.07 | 84.15 | 86.48 | 86.24 | 89.15 | 90.35 | 87.79 |
| EBA | 84.59 | 84.22 | 84.22 | 84.67 | 85.04 | 82.73 | 82.23 | 83.66 | 84.83 |
| FTE | 66.18 | 65.08 | 66.04 | 66.10 | 65.97 | 64.79 | 67.52 | 64.89 | 67.06 |
| SE | 63.07 | 60.71 | 62.98 | 62.71 | 70.51 | 62.29 | 37.66 | 65.00 | 64.07 |
| IR | 52.79 | 52.07 | 52.87 | 52.96 | 51.60 | 50.54 | 56.34 | 39.79 | 53.72 |
| DBT | 40.85 | 40.85 | 40.71 | 41.59 | 39.10 | 39.40 | 47.47 | 41.27 | 42.44 |
| CBT | 36.93 | 45.88 | 37.19 | 37.80 | 35.52 | 31.96 | 42.57 | 34.40 | 36.88 |
| RPE | 32.56 | 32.07 | 32.45 | 33.12 | 30.75 | 44.30 | 38.21 | 32.39 | 22.71 |
| PPT | 27.31 | 25.97 | 27.73 | 28.19 | 27.19 | 27.79 | 29.04 | 41.42 | 29.51 |
| Con | 8.02 | 6.87 | 8.73 | 8.72 | 7.84 | 9.97 | 9.81 | 6.82 | 10.98 |
| Mean (95% CrI) | | | | | | | | | |
| MBE | -1.56 | -1.56 (-2.51, -0.61) | -1.56 (-2.53, -0.58) | -1.49 (-2.66, -0.30) | -1.64 (-2.63, -0.65) | -1.55 (-2.55, -0.55) | -1.38 (-2.25, -0.50) | -1.52 (-2.37, -0.68) | -1.55 (-2.52, -0.60) |
| SE | -1.01 | -1.02 (-1.96, -0.07) | -1.02 (-1.98, -0.06) | -0.99 (-1.99, -0.00) | -1.27 (-2.43, -0.12) | -1.01 (-2.02, -0.01) | -0.43 (-1.46, 0.59) | -0.97 (-1.80, -0.13) | -1.01 (-1.96, -0.05) |
| IR | -0.80 | -0.85 (-1.71, 0.04) | -0.81 (-1.75, 0.11) | -0.79 (-1.67, 0.11) | -0.84 (-1.69, 0.04) | -0.76 (-1.67, 0.15) | -0.71 (-1.48, 0.08) | -0.55 (-1.34, 0.25) | -0.79 (-1.65, 0.08) |
| DBT | -0.56 | -0.62 (-1.79, 0.55) | -0.56 (-1.74, 0.64) | -0.56 (-1.74, 0.63) | -0.57 (-1.75, 0.58) | -0.53 (-1.76, 0.71) | -0.56 (-1.62, 0.49) | -0.55 (-1.58, 0.48) | -0.56 (-1.73, 0.60) |
| CBT | -0.51 | -0.72 (-1.69, 0.25) | -0.51 (-1.37, 0.37) | -0.51 (-1.37, 0.36) | -0.52 (-1.36, 0.34) | -0.40 (-1.46, 0.67) | -0.49 (-1.25, 0.26) | -0.45 (-1.20, 0.30) | -0.46 (-1.31, 0.41) |
| RPE | -0.41 | -0.46 (-1.43, 0.52) | -0.41 (-1.40, 0.58) | -0.41 (-1.38, 0.59) | -0.41 (-1.36, 0.56) | -0.63 (-1.94, 0.67) | -0.41 (-1.28, 0.45) | -0.40 (-1.24, 0.45) | -0.13 (-1.29, 1.03) |
| PPT | -0.36 | -0.39 (-1.20, 0.43) | -0.35 (-1.23, 0.53) | -0.36 (-1.21, 0.48) | -0.38 (-1.22, 0.45) | -0.35 (-1.22, 0.51) | -0.30 (-1.04, 0.44) | -0.57 (-1.31, 0.18) | -0.35 (-1.18, 0.48) |

**Note:** CBT: Cognitive Behavioral Therapy; EBA: Education with Behavioral Activation; RPE: Relaxation and Psychological Education; MBE: Mind Body Exercise; SE: Structured Exercise; FTE: Functional and Targeted Exercise; PPT: Passive Physical Therapy; DBT: Digital and Biofeedback Therapy; IR: Integrated Rehabilitation.

**Table S9** Leave-One-Out sensitivity analysis results of Depression

| Treat-ment | Overall Baseline | Removed Study | | | | | | | | | |
| --- | --- | --- | --- | --- | --- | --- | --- | --- | --- | --- | --- |
|  |  | Study19 | Study5 | Study8 | Study3 | Study1 | Study2 | Study21 | Study13 | Study20 | Study9 |
| SUCRA RANK (%) | | | | | | | | | | | |
| MBE | 94.16 | 79.77 | 93.85 | 92.65 | 93.09 | 91.91 | 93.93 | 94.51 | 93.73 | 95.64 | 95.11 |
| SE | 71.82 | 73.34 | 78.5 | 69.61 | 85.64 | 68.83 | 70.48 | 72.82 | 72.57 | 47.42 | 72.16 |
| IR | 71.73 | 76.62 | 69.31 | 69.81 | 69.48 | 70.12 | 70.42 | 64.99 | 71.85 | 77.75 | 71.22 |
| EBA | 57.87 | 56.62 | 43.41 | 56.02 | 64.21 | 56.08 | 61.78 | 55.22 | 59 | 50.48 | 58.42 |
| CBT | 47.84 | 53.01 | 50.04 | 47.1 | 42.95 | 52.46 | 45.5 | 46.83 | 47.45 | 55.22 | 46.17 |
| RPE | 43.54 | 49.7 | 46.42 | 41 | 38.11 | 46.86 | 40.95 | 43.81 | 35.35 | 51.31 | 41.78 |
| FTE | 42.24 | 33.82 | 40.16 | 40.59 | 44.56 | 41.26 | 57.8 | 40.4 | 43.67 | 42.08 | 45.4 |
| PPT | 34.44 | 36.04 | 35.87 | 33.19 | 32.08 | 34.55 | 24.31 | 44.66 | 36.75 | 37.8 | 34.64 |
| DBT | 29.57 | 32.84 | 32.53 | 43.69 | 24.89 | 30.34 | 27.72 | 29.82 | 30.79 | 33.95 | 28.38 |
| Con | 6.8 | 8.24 | 9.91 | 6.34 | 4.98 | 7.59 | 7.1 | 6.93 | 8.84 | 8.33 | 6.73 |
| Mean (95% CrI) | | | | | | | | | | | |
| MBE | -1.14 | -0.76 (-1.42, -0.08) | -1.14 (-1.74, -0.54) | -1.14 (-1.75, -0.53) | -1.24 (-1.80, -0.69) | -1.15 (-1.78, -0.51) | -1.21 (-1.81, -0.60) | -1.13 (-1.72, -0.53) | -1.14 (-1.75, -0.52) | -1.04 (-1.58, -0.49) | -1.27 (-2.01, -0.55) |
| SE | -0.77 | -0.67 (-1.20, -0.13) | -0.86 (-1.50, -0.23) | -0.77 (-1.36, -0.18) | -1.10 (-1.75, -0.45) | -0.77 (-1.38, -0.15) | -0.81 (-1.39, -0.23) | -0.75 (-1.33, -0.18) | -0.77 (-1.36, -0.17) | -0.39 (-1.03, 0.22) | -0.80 (-1.41, -0.20) |
| IR | -0.74 | -0.68 (-1.18, -0.16) | -0.67 (-1.25, -0.07) | -0.75 (-1.30, -0.18) | -0.81 (-1.30, -0.29) | -0.76 (-1.34, -0.17) | -0.79 (-1.36, -0.23) | -0.64 (-1.20, -0.08) | -0.73 (-1.29, -0.16) | -0.68 (-1.17, -0.18) | -0.76 (-1.32, -0.19) |
| CBT | -0.48 | -0.46 (-0.93, 0.02) | -0.46 (-1.01, 0.07) | -0.50 (-1.06, 0.04) | -0.49 (-0.98, -0.01) | -0.57 (-1.25, 0.11) | -0.49 (-1.02, 0.04) | -0.46 (-0.98, 0.07) | -0.46 (-1.00, 0.08) | -0.46 (-0.93, 0.01) | -0.48 (-1.02, 0.05) |
| RPE | -0.43 | -0.42 (-0.96, 0.13) | -0.42 (-1.04, 0.20) | -0.43 (-1.04, 0.19) | -0.43 (-0.98, 0.13) | -0.49 (-1.33, 0.35) | -0.43 (-1.03, 0.17) | -0.42 (-1.02, 0.18) | -0.30 (-1.05, 0.46) | -0.42 (-0.95, 0.10) | -0.43 (-1.03, 0.19) |
| PPT | -0.34 | -0.30 (-0.80, 0.18) | -0.31 (-0.85, 0.23) | -0.35 (-0.89, 0.19) | -0.38 (-0.86, 0.11) | -0.37 (-0.93, 0.19) | -0.23 (-0.78, 0.31) | -0.44 (-0.98, 0.09) | -0.35 (-0.89, 0.19) | -0.30 (-0.78, 0.17) | -0.36 (-0.90, 0.17) |
| DBT | -0.25 | -0.23 (-0.92, 0.44) | -0.24 (-0.99, 0.51) | -0.44 (-1.48, 0.60) | -0.24 (-0.93, 0.44) | -0.27 (-1.06, 0.53) | -0.24 (-0.98, 0.50) | -0.24 (-0.98, 0.49) | -0.24 (-0.99, 0.52) | -0.24 (-0.90, 0.41) | -0.24 (-0.99, 0.49) |

**Note:** CBT: Cognitive Behavioral Therapy; EBA: Education with Behavioral Activation; RPE: Relaxation and Psychological Education; MBE: Mind Body Exercise; SE: Structured Exercise; FTE: Functional and Targeted Exercise; PPT: Passive Physical Therapy; DBT: Digital and Biofeedback Therapy; IR: Integrated Rehabilitation.

**Table S10** Leave-One-Out sensitivity analysis results of Anxiety

| Treat-ment | Overall Baseline | Removed Study | | | | | | | |
| --- | --- | --- | --- | --- | --- | --- | --- | --- | --- |
|  |  | Study19 | Study12 | Study8 | Study1 | Study2 | Study24 | Study20 | Study9 |
| SUCRA RANK (%) | | | | | | | | | |
| MBE | 98.77 | 96.84 | 98.34 | 98.29 | 98.15 | 99.45 | 98.97 | 99.13 | 97.89 |
| EBA | 79.09 | 78.53 | 74.84 | 77.29 | 76.92 | 84.44 | 77.79 | 77.70 | 78.29 |
| SE | 75.26 | 74.68 | 74.45 | 72.92 | 73.03 | 74.28 | 76.26 | 61.64 | 74.87 |
| CBT | 51.95 | 53.79 | 52.60 | 51.09 | 49.86 | 47.00 | 52.00 | 56.91 | 52.63 |
| IR | 50.61 | 49.92 | 51.94 | 48.11 | 49.24 | 50.45 | 42.44 | 52.41 | 50.39 |
| RPE | 48.01 | 50.30 | 48.24 | 45.75 | 53.21 | 41.97 | 49.15 | 52.99 | 48.33 |
| DBT | 31.73 | 33.49 | 32.66 | 46.48 | 32.36 | 25.44 | 31.80 | 34.40 | 32.63 |
| FTE | 26.79 | 22.30 | 26.90 | 24.98 | 26.93 | 57.26 | 24.28 | 24.42 | 25.74 |
| PPT | 19.06 | 19.23 | 20.61 | 17.84 | 20.18 | 4.00 | 28.45 | 19.17 | 19.63 |
| Con | 18.73 | 20.92 | 19.40 | 17.24 | 20.12 | 15.71 | 18.86 | 21.24 | 19.59 |
| Mean (95% CrI) | | | | | | | | | |
| MBE | -1.38 | -1.19 (-1.90, -0.49) | -1.38 (-1.98, -0.79) | -1.39 (-1.98, -0.79) | -1.38 (-2.00, -0.77) | -1.53 (-2.00, -1.07) | -1.37 (-1.93, -0.79) | -1.31 (-1.87, -0.75) | -1.33 (-2.03, -0.62) |
| SE | -0.69 | -0.64 (-1.21, -0.06) | -0.68 (-1.27, -0.08) | -0.69 (-1.26, -0.11) | -0.69 (-1.29, -0.09) | -0.76 (-1.20, -0.33) | -0.67 (-1.21, -0.12) | -0.42 (-1.09, 0.23) | -0.68 (-1.27, -0.10) |
| IR | -0.35 | -0.34 (-0.83, 0.17) | -0.35 (-0.86, 0.19) | -0.37 (-0.88, 0.16) | -0.34 (-0.99, 0.33) | -0.38 (-0.77, 0.01) | -0.32 (-0.82, 0.18) | -0.34 (-0.81, 0.14) | -0.35 (-0.86, 0.17) |
| DBT | -0.33 | -0.29 (-0.81, 0.25) | -0.34 (-0.90, 0.24) | -0.33 (-0.86, 0.21) | -0.32 (-0.90, 0.27) | -0.43 (-0.87, 0.01) | -0.22 (-0.76, 0.33) | -0.29 (-0.79, 0.24) | -0.32 (-0.86, 0.25) |
| CBT | -0.31 | -0.31 (-0.88, 0.28) | -0.31 (-0.90, 0.31) | -0.32 (-0.90, 0.28) | -0.40 (-1.23, 0.44) | -0.32 (-0.77, 0.13) | -0.30 (-0.87, 0.26) | -0.31 (-0.86, 0.25) | -0.31 (-0.90, 0.30) |
| RPE | -0.11 | -0.11 (-0.84, 0.61) | -0.11 (-0.87, 0.63) | -0.34 (-1.39, 0.71) | -0.11 (-0.89, 0.65) | -0.10 (-0.69, 0.49) | -0.10 (-0.81, 0.60) | -0.10 (-0.81, 0.61) | -0.11 (-0.86, 0.64) |
| PPT | 0.02 | 0.04 (-0.46, 0.55) | 0.01 (-0.52, 0.54) | 0.01 (-0.50, 0.53) | 0.02 (-0.53, 0.57) | 0.24 (-0.19, 0.67) | -0.08 (-0.60, 0.43) | 0.04 (-0.45, 0.53) | 0.02 (-0.50, 0.54) |

**Note:** CBT: Cognitive Behavioral Therapy; EBA: Education with Behavioral Activation; RPE: Relaxation and Psychological Education; MBE: Mind Body Exercise; SE: Structured Exercise; FTE: Functional and Targeted Exercise; PPT: Passive Physical Therapy; DBT: Digital and Biofeedback Therapy; IR: Integrated Rehabilitation.

**Table S11** Pooled Effect Sizes and Subgroup Heterogeneity (τ) Across Three Outcomes Using Different Meta-Analytic Models

| model | Group | Effect Size (95% CI) | Prob Positive | τ | Mean Diff (95% CI) | Prob Meaningful Diff |
| --- | --- | --- | --- | --- | --- | --- |
| Pain  (Random) | Subgroup LQ | -0.029 (-0.606, 0.552) | 0.457 | 1.082 | 0.008 (-0.613, 0.627) | 0.746 |
|  | Subgroup HQ | -0.053 (-0.534, 0.42) | 0.405 | 0.727 |  |  |
|  | Subgroup ≥ 45 | -0.211 (-0.69, 0.282) | 0.17 | 0.583 | -0.085 (-0.715, 0.535) | 0.753 |
|  | Subgroup < 45 | -0.079 (-0.71, 0.564) | 0.395 | 0.931 |  |  |
|  | Subgroup LD | 0.369 (-0.117, 0.828) | 0.945 | 0.595 | 0.399 (-0.22, 1.024) | 0.89 |
|  | Subgroup HD | -0.247 (-0.762, 0.276) | 0.17 | 1 |  |  |
|  | Subgroup LB | 0.087 (-0.405, 0.572) | 0.642 | 0.895 | 0.15 (-0.537, 0.812) | 0.78 |
|  | Subgroup HB | -0.178 (-0.998, 0.635) | 0.324 | 1.193 |  |  |
|  | Subgroup ST | -0.297 (-1.037, 0.501) | 0.183 | 0.711 | -0.176 (-0.884, 0.551) | 0.802 |
|  | Subgroup LT | 0.012 (-0.437, 0.457) | 0.52 | 0.981 |  |  |
|  | Subgroup LF | 0.257 (-0.194, 0.703) | 0.888 | 0.621 | -0.174(-0.786, 0.446) | 0.785 |
|  | Subgroup HF | -0.015 (-0.621, 0.633) | 0.469 | 1.108 |  |  |
|  | Subgroup PL | 0.231 (-0.530, 0.997) | 0.728 | 1.172 | 0.336 (-0.308, 0.984) | 0.861 |
|  | Subgroup TL | -0.290 (-0.712, 0.139) | 0.081 | 0.752 |  |  |
| Depression(Random) | Subgroup LQ | 0.041 (-0.434, 0.521) | 0.572 | 0.83 | 0.038 (-0.453, 0.51) | 0.674 |
|  | Subgroup HQ | 0.03 (-0.19, 0.228) | 0.641 | 0.21 |  |  |
|  | Subgroup ≥ 45 | -0.002 (-0.22, 0.21) | 0.499 | 0.15 | 0.026 (-0.516, 0.564) | 0.707 |
|  | Subgroup < 45 | -0.041 (-0.6, 0.53) | 0.435 | 0.843 |  |  |
|  | Subgroup LD | 0.194 (0.008, 0.398) | 0.979 | 0.117 | 0.209 (-0.291, 0.704) | 0.776 |
|  | Subgroup HD | -0.051 (-0.436, 0.337) | 0.396 | 0.749 |  |  |
|  | Subgroup LB | 0.171 (-0.126, 0.478) | 0.885 | 0.491 | 0.262 (-0.279, 0.795) | 0.818 |
|  | Subgroup HB | -0.184 (-0.922, 0.528) | 0.296 | 0.957 |  |  |
|  | Subgroup ST | 0.004 (-0.519, 0.541) | 0.506 | 0.388 | -0.02 (-0.61, 0.572) | 0.74 |
|  | Subgroup LT | 0.037 (-0.276, 0.345) | 0.604 | 0.67 |  |  |
|  | Subgroup LF | 0.140 (-0.029, 0.334) | 0.951 | 0.118 | -0.006 (-0.452, 0.426) | 0.638 |
|  | Subgroup HF | 0.167 ( -0.251, 0.585) | 0.805 | 0.639 |  |  |
|  | Subgroup PL | 0.219 (-0.302, 0.741) | 0.819 | 0.660 | 0.255 (-0.271, 0.763) | 0.824 |
|  | Subgroup TL | -0.105 (-0.493, 0.270) | 0.282 | 0.639 |  |  |
| Anxiety  (Random) | Subgroup LQ | -0.063 (-0.521, 0.402) | 0.38 | 0.826 | 0.124 (-0.366, 0.634) | 0.718 |
|  | Subgroup HQ | -0.208 (-0.484, 0.048) | 0.051 | 0.336 |  |  |
|  | Subgroup ≥ 45 | -0.001 (-0.424, 0.378) | 0.51 | 0.434 | 0.135 (-0.415, 0.708) | 0.746 |
|  | Subgroup < 45 | -0.165 (-0.71, 0.392) | 0.26 | 0.803 |  |  |
|  | Subgroup LD | 0.107 (-0.134, 0.317) | 0.843 | 0.179 | 0.206 (-0.321, 0.712) | 0.78 |
|  | Subgroup HD | -0.231 (-0.64, 0.192) | 0.132 | 0.792 |  |  |
|  | Subgroup LB | -0.002 (-0.335, 0.322) | 0.503 | 0.56 | 0.043 (-0.526, 0.63) | 0.731 |
|  | Subgroup HB | -0.057 (-0.846, 0.777) | 0.434 | 1.076 |  |  |
|  | Subgroup ST | -0.037 (-0.858, 0.783) | 0.456 | 0.827 | 0.071 (-0.53, 0.676) | 0.755 |
|  | Subgroup LT | -0.153 (-0.458, 0.156) | 0.152 | 0.647 |  |  |
|  | Subgroup LF | 0.042 (-0.136, 0.200) | 0.706 | 0.102 | 0.007 (-0.43, 0.453) | 0.641 |
|  | Subgroup HF | 0.000 (-0.453, 0.444) | 0.498 | 0.691 |  |  |
|  | Subgroup PL | -0.018 (-0.588, 0.542) | 0.476 | 0.794 | 0.208 (-0.317, 0.719) | 0.787 |
|  | Subgroup TL | -0.264 (-0.618, 0.116) | 0.075 | 0.601 |  |  |

**Note:** LQ: Low quality; HQ: High quality; ≥45: ≥ 45 years; <45: < 45 years; LD: Low Dose; HD: High Dose; LB: Low baseline pain; HB: High baseline pain; ST: Short-term intervention; LT: Long-term intervention; LF: Low frequency; HF: High frequency; PL: Patient-led; TL: Therapist-led.

**Table S12** Summary of Bayesian Network Meta-regression Results for Pain

| Covariate | Model Type | k | Intercept  [CrI] | Beta | CrI | Tau2  Resid | I2  Resid | R2 | Delta  LOO |
| --- | --- | --- | --- | --- | --- | --- | --- | --- | --- |
| Mean age | Linear | 27 | -0.868 [-3.604,1.842] | 0.016 | [-0.045,0.077] | 0.8411 | 92.60% | 8% | -9.8 |
|  | Quadratic term | 27 | 6.793 [-2.819,16.386] | -0.341  Poly: 0.004 | [-0.776,0.091] | 0.8465 | 92.60% | 7.4% | -14.8 |
| Intervention does | Linear | 27 | 0.207 [-0.336,0.784] | -0.02 | [-0.045,0.004] | 0.8298 | 92.50% | 9.2% | -15.4 |
|  | Quadratic term | 27 | 0.543 [-0.145,1.252] | -0.066  Poly: 0.001 | [-0.124,-0.009] | 0.7988 | 92.20% | 12.6% | -19.9 |
| Baseline pain | Linear | 27 | 0.583 [-0.744,2] | -0.136 | [-0.38,0.098] | 0.911 | 93.10% | 0.3% | -16 |
|  | Quadratic term | 27 | 0.459 [-1.227,2.209] | -0.062  Poly: -0.009 | [-0.618,0.506] | 0.927 | 93.20% | 0% | -15.7 |
| Intervention times | Linear | 27 | 0.544 [-1.082,2.097] | -0.196 | [-0.621,0.246] | 0.7905 | 92.10% | 13.5% | -13 |
|  | Quadratic term | 27 | 0.596 [-0.933,2.134] | -0.042  Poly: -0.046 | [-0.61,0.525] | 0.7944 | 92.10% | 13.1% | -9.7 |
| Intervention frequency | Linear | 27 | 0.049 [-0.467,0.553] | -0.046 | [-0.128,0.038] | 0.8067 | 92.30% | 11.7% | -12.3 |
|  | Quadratic term | 27 | -0.317 [-0.989,0.429] | 0.11  Poly: -0.008 | [-0.121,0.329] | 0.7477 | 91.70% | 18.2% | -9.2 |
| Therapist involvement | Categorical | 27 | -0.258 [-0.699,0.189] | 0.173 | [-0.294,0.622] | 0.8053 | 92.20% | 11.9% | -14.1 |
| Intervention methods | Categorical | 27 | -0.126 [-0.496,0.25] | -0.138 | [-0.567,0.307] | 0.799 | 92.20% | 12.6% | -11.2 |
| Research quality | Categorical | 27 | -0.248 [-0.643,0.165] | 0.197 | [-0.273,0.648] | 0.7957 | 92.20% | 12.9% | -13.3 |

**Note:** k: number of studies; CrI: 95% Bayesian credibility interval; Tau2: between-group variance of residuals; I^2^: heterogeneity of residuals; R^2^: proportion of between-group variance explained by covariates; Delta LOO: difference in leave-one-out cross-validation compared with the baseline model (M0); Linear: refers to the first-order term of a continuous variable; Quadratic term: refers to a quadratic polynomial model (y = β₀ + β₁X + β₂X²); Categorical: denotes moderation analysis for discrete variables; Poly: denotes the coefficient of the quadratic term (β₂); Beta: for linear models, Beta represents the regression coefficient of the covariate; for quadratic models, Beta denotes the coefficient of the linear term. If the 95% CrI of a covariate does not include zero, that covariate is considered a significant moderator.

**Table S13** Summary of Bayesian Network Meta-regression Results for Depression

| Covariate | Model Type | k | Intercept  [CrI] | Beta | CrI | Tau2  Resid | I2  Resid | R2 | Delta  LOO |
| --- | --- | --- | --- | --- | --- | --- | --- | --- | --- |
| Mean age | Linear | 27 | -0.961 [-3.179,1.214] | 0.017 | [-0.032,0.066] | 0.3646 | 83.30% | 6.2% | -2.8 |
|  | Quadratic term | 27 | -0.745 [-9.89,8.326] | 0.007  Poly: 0 | [-0.408,0.42] | 0.3634 | 83.20% | 6.5% | -2.7 |
| Intervention does | Linear | 27 | -0.147 [-0.56,0.268] | -0.004 | [-0.022,0.015] | 0.3688 | 83.40% | 5.1% | -3.5 |
|  | Quadratic term | 27 | -0.119 [-0.661,0.427] | -0.007  Poly: 0 | [-0.057,0.042] | 0.3811 | 83.90% | 1.9% | -3.3 |
| Baseline pain | Linear | 27 | -0.123 [-1.161,0.929] | -0.016 | [-0.201,0.17] | 0.3634 | 83.20% | 6.5% | -1.8 |
|  | Quadratic term | 27 | -0.208 [-1.765,1.348] | 0.025  Poly: -0.005 | [-0.503,0.549] | 0.3649 | 83.30% | 6.1% | -1.8 |
| Intervention times | Linear | 27 | 0.652 [-0.615,1.926] | -0.24 | [-0.583,0.107] | 0.3296 | 81.80% | 15.2% | -6.2 |
|  | Quadratic term | 27 | 0.485 [-0.829,1.768] | -0.019  Poly: -0.047 | [-0.582,0.545] | 0.3329 | 82% | 14.3% | -6.4 |
| Intervention frequency | Linear | 27 | -0.016 [-0.389,0.365] | -0.043 | [-0.102,0.015] | 0.3298 | 81.80% | 15.1% | -3.7 |
|  | Quadratic term | 27 | -0.184 [-0.719,0.351] | 0.026 Poly: -0.003 | [-0.15,0.208] | 0.3318 | 81.90% | 14.6% | -1.9 |
| Therapist involvement | Categorical | 27 | -0.317 [-0.656,0.03] | 0.179 | [-0.218,0.561] | 0.3395 | 82.30% | 12.6% | -4 |
| Intervention methods | Categorical | 27 | -0.122 [-0.396,0.152] | -0.321 | [-0.696,0.089] | 0.3048 | 80.60% | 21.6% | -5.6 |
| Research quality | Categorical | 27 | -0.321 [-0.614,-0.021] | 0.258 | [-0.135,0.643] | 0.317 | 81.20% | 18.4% | -5 |

**Note:** k: number of studies; CrI: 95% Bayesian credibility interval; Tau2: between-group variance of residuals; I^2^: heterogeneity of residuals; R^2^: proportion of between-group variance explained by covariates; Delta LOO: difference in leave-one-out cross-validation compared with the baseline model (M0); Linear: refers to the first-order term of a continuous variable; Quadratic term: refers to a quadratic polynomial model (y = β₀ + β₁X + β₂X²); Categorical: denotes moderation analysis for discrete variables; Poly: denotes the coefficient of the quadratic term (β₂); Beta: for linear models, Beta represents the regression coefficient of the covariate; for quadratic models, Beta denotes the coefficient of the linear term. If the 95% CrI of a covariate does not include zero, that covariate is considered a significant moderator.

**Table S14** Summary of Bayesian Network Meta-regression Results for Anxiety

| Covariate | Model Type | k | Intercept  [CrI] | Beta | CrI | Tau2  Resid | I2  Resid | R2 | Delta  LOO |
| --- | --- | --- | --- | --- | --- | --- | --- | --- | --- |
| Mean age | Linear | 27 | -0.727 [-3.225,1.738] | 0.016 | [-0.038,0.073] | 0.4989 | 86.90% | 3.2% | -8 |
|  | Quadratic term | 27 | -1.455 [-10.75,8.128] | 0.05  Poly: 0 | [-0.382,0.471] | 0.5012 | 86.90% | 2.8% | -8.7 |
| Intervention does | Linear | 27 | 0.122 [-0.342,0.58] | -0.007 | [-0.028,0.013] | 0.4893 | 86.70% | 5.1% | -10.2 |
|  | Quadratic term | 27 | 0.232 [-0.361,0.833] | -0.023  Poly: 0 | [-0.075,0.03] | 0.5029 | 87% | 2.5% | -9.3 |
| Baseline pain | Linear | 27 | -0.004 [-1.158,1.15] | -0.001 | [-0.206,0.21] | 0.4872 | 86.60% | 5.50% | -8.2 |
|  | Quadratic term | 27 | 0.046 [-1.539,1.652] | -0.027  Poly: 0.003 | [-0.56,0.515] | 0.4925 | 86.70% | 4.5% | -10.4 |
| Intervention times | Linear | 27 | 1.303 [-0.049,2.603] | -0.365 | [-0.719,0.005] | 0.3805 | 83.50% | 26.2% | -12.7 |
|  | Quadratic term | 27 | 1.103 [-0.236,2.474] | -0.008  Poly: -0.082 | [-0.58,0.568] | 0.3663 | 83% | 29% | -12.6 |
| Intervention frequency | Linear | 27 | 0.17 [-0.253,0.579] | -0.04 | [-0.103,0.024] | 0.4522 | 85.70% | 12.3% | -7.9 |
|  | Quadratic term | 27 | 0.053 [-0.533,0.661] | 0.01  Poly: -0.003 | [-0.185,0.204] | 0.4733 | 86.30% | 8.2% | -7.1 |
| Therapist involvement | Categorical | 27 | -0.193 [-0.545,0.185] | 0.31 | [-0.121,0.714] | 0.4129 | 84.60% | 19.9% | -8.9 |
| Intervention methods | Categorical | 27 | 0.115 [-0.177,0.376] | -0.469 | [-0.871,-0.049] | 0.3341 | 81.60% | 35.2% | -10 |
| Research quality | Categorical | 27 | -0.148 [-0.471,0.207] | 0.32 | [-0.118,0.731] | 0.4178 | 84.70% | 19% | -11.1 |

**Note:** k: number of studies; CrI: 95% Bayesian credibility interval; Tau2: between-group variance of residuals; I^2^: heterogeneity of residuals; R^2^: proportion of between-group variance explained by covariates; Delta LOO: difference in leave-one-out cross-validation compared with the baseline model (M0); Linear: refers to the first-order term of a continuous variable; Quadratic term: refers to a quadratic polynomial model (y = β₀ + β₁X + β₂X²); Categorical: denotes moderation analysis for discrete variables; Poly: denotes the coefficient of the quadratic term (β₂); Beta: for linear models, Beta represents the regression coefficient of the covariate; for quadratic models, Beta denotes the coefficient of the linear term. If the 95% CrI of a covariate does not include zero, that covariate is considered a significant moderator.
