# Supplementary material for: Comparative effectiveness of non-pharmacological interventions for depression and anxiety in chronic low back pain: a Bayesian network meta-analysis of randomized controlled trials
Source: Front Public Health. 2026 Apr 20;14:1765762. doi: 10.3389/fpubh.2026.1765762 (PMC13137130; doi:10.3389/fpubh.2026.1765762)
Supplement: Supplementary file 2 [file Data_Sheet_1.pdf]

Pain

Download Report | Reset

Comparison	Number of Studies	Within-study bias	Reporting bias	Indirectness	Imprecision	Heterogeneity	Incoherence	Confidence rating	Reason(s) for downgrading
Mixed evidence									
CBT vs Con	3	Some concerns	Some concerns	No concerns	Some concerns	Some concerns	No concerns	Moderate	
CBT vs DBT	1	Some concerns	Some concerns	No concerns	Major concerns	No concerns	No concerns	Low	Imprecision
CBT vs IR	1	No concerns	Some concerns	No concerns	Major concerns	No concerns	No concerns	Low	Imprecision
CBT vs RPE	1	Some concerns	Some concerns	No concerns	Major concerns	No concerns	No concerns	Low	Imprecision
Con vs DBT	2	Some concerns	Some concerns	No concerns	Major concerns	No concerns	No concerns	Low	Imprecision
Con vs IR	1	No concerns	Some concerns	No concerns	No concerns	Major concerns	Some concerns	Low	Heterogeneity
Con vs MBE	2	No concerns	Some concerns	No concerns	No concerns	Some concerns	No concerns	Moderate	
Con vs PPT	3	Some concerns	Some concerns	No concerns	Some concerns	Some concerns	No concerns	Moderate	
Con vs RPE	3	Some concerns	Some concerns	No concerns	Some concerns	Some concerns	No concerns	Moderate	
Con vs SE	2	No concerns	Some concerns	No concerns	No concerns	Major concerns	No concerns	Low	Heterogeneity
EBA vs FTE	1	No concerns	Some concerns	No concerns	Major concerns	No concerns	No concerns	Low	Imprecision
EBA vs IR	1	No concerns	Some concerns	No concerns	Some concerns	Some concerns	Some concerns	Moderate	
EBA vs SE	1	No concerns	Some concerns	No concerns	Major concerns	No concerns	No concerns	Low	Imprecision
FTE vs IR	2	No concerns	Some concerns	No concerns	Major concerns	No concerns	No concerns	Low	Imprecision
FTE vs MBE	1	No concerns	Some concerns	No concerns	Major concerns	No concerns	No concerns	Low	Imprecision
FTE vs PPT	1	No concerns	Some concerns	No concerns	Some concerns	Some concerns	No concerns	Moderate	
IR vs PPT	3	Some concerns	Some concerns	No concerns	Some concerns	Some concerns	No concerns	Moderate	
MBE vs SE	1	No concerns	Some concerns	No concerns	Some concerns	Some concerns	No concerns	Moderate	
Indirect evidence									
CBT vs EBA	--	No concerns	Some concerns	No concerns	Some concerns	Some concerns	Major concerns	Low	Incoherence
CBT vs FTE	--	No concerns	Some concerns	No concerns	Major concerns	No concerns	Major concerns	Very low	Imprecision, Incoherence
CBT vs MBE	--	Some concerns	Some concerns	No concerns	Some concerns	Some concerns	Major concerns	Low	Incoherence
CBT vs PPT	--	Some concerns	Some concerns	No concerns	Major concerns	No concerns	Major concerns	Very low	Imprecision, Incoherence
CBT vs SE	--	Some concerns	Some concerns	No concerns	Major concerns	No concerns	Major concerns	Very low	Imprecision, Incoherence
Con vs EBA	--	No concerns	Some concerns	No concerns	No concerns	Some concerns	Major concerns	Low	Incoherence
Con vs FTE	--	No concerns	Some concerns	No concerns	No concerns	Major concerns	Major concerns	Very low	Heterogeneity, Incoherence
DBT vs EBA	--	Some concerns	Some concerns	No concerns	Major concerns	No concerns	Major concerns	Very low	Imprecision, Incoherence
DBT vs FTE	--	Some concerns	Some concerns	No concerns	Major concerns	No concerns	Major concerns	Very low	Imprecision, Incoherence
DBT vs IR	--	Some concerns	Some concerns	No concerns	Major concerns	No concerns	Major concerns	Very low	Imprecision, Incoherence
DBT vs MBE	--	Some concerns	Some concerns	No concerns	Some concerns	Some concerns	Major concerns	Low	Incoherence
DBT vs PPT	--	Some concerns	Some concerns	No concerns	Major concerns	No concerns	Major concerns	Very low	Imprecision, Incoherence
DBT vs RPE	--	Some concerns	Some concerns	No concerns	Major concerns	No concerns	Major concerns	Very low	Imprecision, Incoherence
DBT vs SE	--	Some concerns	Some concerns	No concerns	Major concerns	No concerns	Major concerns	Very low	Imprecision, Incoherence
EBA vs MBE	--	No concerns	Some concerns	No concerns	Major concerns	No concerns	Major concerns	Very low	Imprecision, Incoherence
EBA vs PPT	--	No concerns	Some concerns	No concerns	Some concerns	Some concerns	Major concerns	Low	Incoherence
EBA vs RPE	--	No concerns	Some concerns	No concerns	Some concerns	Some concerns	Major concerns	Low	Incoherence
FTE vs RPE	--	No concerns	Some concerns	No concerns	Major concerns	No concerns	Major concerns	Very low	Imprecision, Incoherence
FTE vs SE	--	No concerns	Some concerns	No concerns	Major concerns	No concerns	Major concerns	Very low	Imprecision, Incoherence
IR vs MBE	--	No concerns	Some concerns	No concerns	Some concerns	Some concerns	Major concerns	Low	Incoherence
IR vs RPE	--	No concerns	Some concerns	No concerns	Major concerns	No concerns	Major concerns	Very low	Imprecision, Incoherence
IR vs SE	--	No concerns	Some concerns	No concerns	Major concerns	No concerns	Major concerns	Very low	Imprecision, Incoherence
MBE vs PPT	--	No concerns	Some concerns	No concerns	No concerns	Major concerns	Major concerns	Very low	Heterogeneity, Incoherence
MBE vs RPE	--	No concerns	Some concerns	No concerns	Some concerns	Some concerns	Major concerns	Low	Incoherence
PPT vs RPE	--	Some concerns	Some concerns	No concerns	Major concerns	No concerns	Major concerns	Very low	Imprecision, Incoherence
PPT vs SE	--	Some concerns	Some concerns	No concerns	Some concerns	Some concerns	Major concerns	Low	Incoherence
RPE vs SE	--	No concerns	Some concerns	No concerns	Major concerns	No concerns	Major concerns	Very low	Imprecision, Incoherence

Figure S1 CINeMA Ratings: Pain assessed at the comparison average ROB and indirectness

Note: CBT: Cognitive Behavioral Therapy; EBA: Education with Behavioral Activation; RPE: Relaxation and Psychological Education; MBE: Mind Body Exercise; SE: Structured Exercise; FTE: Functional and Targeted Exercise; PPT: Passive Physical Therapy; DBT: Digital and Biofeedback Therapy; IR: Integrated Rehabilitation.

Depression

Download Report Reset

Comparison	Number of Studies	Within-study bias	Reporting bias	Indirectness	Imprecision	Heterogeneity	Incoherence	Confidence rating	Reason(s) for downgrading
Mixed evidence									
CBT vs Con	3	Some concerns	Some concerns	No concerns	No concerns	Major concerns	No concerns	Low	Heterogeneity
CBT vs DBT	1	Some concerns	Some concerns	No concerns	Major concerns	No concerns	No concerns	Low	Imprecision
CBT vs IR	1	No concerns	Some concerns	No concerns	Some concerns	Some concerns	No concerns	Moderate	
CBT vs RPE	1	No concerns	Some concerns	No concerns	Major concerns	No concerns	No concerns	Low	Imprecision
Con vs DBT	2	Some concerns	Some concerns	No concerns	Some concerns	Some concerns	No concerns	Moderate	
Con vs IR	1	No concerns	Some concerns	No concerns	No concerns	Some concerns	No concerns	Moderate	
Con vs MBE	2	No concerns	Some concerns	No concerns	No concerns	No concerns	No concerns	Moderate	
Con vs PPT	3	Some concerns	Some concerns	No concerns	Some concerns	Some concerns	No concerns	Moderate	
Con vs RPE	3	Some concerns	Some concerns	No concerns	Some concerns	Some concerns	No concerns	Moderate	
Con vs SE	2	No concerns	Some concerns	No concerns	No concerns	Some concerns	No concerns	Moderate	
EBA vs FTE	1	No concerns	Some concerns	No concerns	Major concerns	No concerns	No concerns	Low	Imprecision
EBA vs IR	1	No concerns	Some concerns	No concerns	Major concerns	No concerns	No concerns	Low	Imprecision
EBA vs SE	1	No concerns	Some concerns	No concerns	Major concerns	No concerns	No concerns	Low	Imprecision
FTE vs IR	2	No concerns	Some concerns	No concerns	Some concerns	Some concerns	No concerns	Moderate	
FTE vs MBE	1	No concerns	Some concerns	No concerns	No concerns	Some concerns	No concerns	Moderate	
FTE vs PPT	1	No concerns	Some concerns	No concerns	Major concerns	No concerns	No concerns	Low	Imprecision
IR vs PPT	3	Some concerns	Some concerns	No concerns	Some concerns	Some concerns	No concerns	Moderate	
MBE vs SE	1	No concerns	Some concerns	No concerns	Some concerns	Some concerns	No concerns	Moderate	
Indirect evidence									
CBT vs EBA	--	No concerns	Some concerns	No concerns	Major concerns	No concerns	Major concerns	Very low	Imprecision, Incoherence
CBT vs FTE	--	No concerns	Some concerns	No concerns	Major concerns	No concerns	Major concerns	Very low	Imprecision, Incoherence
CBT vs MBE	--	Some concerns	Some concerns	No concerns	Some concerns	Some concerns	Major concerns	Low	Incoherence
CBT vs PPT	--	Some concerns	Some concerns	No concerns	Major concerns	No concerns	Major concerns	Very low	Imprecision, Incoherence
CBT vs SE	--	Some concerns	Some concerns	No concerns	Some concerns	Some concerns	Major concerns	Low	Incoherence
Con vs EBA	--	No concerns	Some concerns	No concerns	Some concerns	Some concerns	Major concerns	Low	Incoherence
Con vs FTE	--	No concerns	Some concerns	No concerns	Some concerns	Some concerns	Major concerns	Low	Incoherence
DBT vs EBA	--	Some concerns	Some concerns	No concerns	Major concerns	No concerns	Major concerns	Very low	Imprecision, Incoherence
DBT vs FTE	--	Some concerns	Some concerns	No concerns	Major concerns	No concerns	Major concerns	Very low	Imprecision, Incoherence
DBT vs IR	--	Some concerns	Some concerns	No concerns	Some concerns	Some concerns	Major concerns	Low	Incoherence
DBT vs MBE	--	Some concerns	Some concerns	No concerns	Some concerns	No concerns	Major concerns	Low	Incoherence
DBT vs PPT	--	Some concerns	Some concerns	No concerns	Major concerns	No concerns	Major concerns	Very low	Imprecision, Incoherence
DBT vs RPE	--	Some concerns	Some concerns	No concerns	Major concerns	No concerns	Major concerns	Very low	Imprecision, Incoherence
DBT vs SE	--	Some concerns	Some concerns	No concerns	Some concerns	Some concerns	Major concerns	Low	Incoherence
EBA vs MBE	--	No concerns	Some concerns	No concerns	Some concerns	Some concerns	Major concerns	Low	Incoherence
EBA vs PPT	--	No concerns	Some concerns	No concerns	Major concerns	No concerns	Major concerns	Very low	Imprecision, Incoherence
EBA vs RPE	--	No concerns	Some concerns	No concerns	Major concerns	No concerns	Major concerns	Very low	Imprecision, Incoherence
FTE vs RPE	--	No concerns	Some concerns	No concerns	Major concerns	No concerns	Major concerns	Very low	Imprecision, Incoherence
FTE vs SE	--	No concerns	Some concerns	No concerns	Some concerns	Some concerns	Major concerns	Low	Incoherence
IR vs MBE	--	No concerns	Some concerns	No concerns	Some concerns	Some concerns	Major concerns	Low	Incoherence
IR vs RPE	--	No concerns	Some concerns	No concerns	Some concerns	Some concerns	Major concerns	Low	Incoherence
IR vs SE	--	No concerns	Some concerns	No concerns	Major concerns	No concerns	Major concerns	Very low	Imprecision, Incoherence
MBE vs PPT	--	No concerns	Some concerns	No concerns	No concerns	Some concerns	Major concerns	Low	Incoherence
MBE vs RPE	--	Some concerns	Some concerns	No concerns	Some concerns	Some concerns	Major concerns	Low	Incoherence
PPT vs RPE	--	Some concerns	Some concerns	No concerns	Major concerns	No concerns	Major concerns	Very low	Imprecision, Incoherence
PPT vs SE	--	No concerns	Some concerns	No concerns	Some concerns	Some concerns	Major concerns	Low	Incoherence
RPE vs SE	--	Some concerns	Some concerns	No concerns	Some concerns	Some concerns	Major concerns	Low	Incoherence

Figure S2 CINeMA Ratings: Depression assessed at the comparison average ROB and indirectness

Note: CBT: Cognitive Behavioral Therapy; EBA: Education with Behavioral Activation; RPE: Relaxation and Psychological Education; MBE: Mind Body Exercise; SE: Structured Exercise; FTE: Functional and Targeted Exercise; PPT: Passive Physical Therapy; DBT: Digital and Biofeedback Therapy; IR: Integrated Rehabilitation.

Anxiety

Download Report | Reset

Comparison	Number of Studies	Within-study bias	Reporting bias	Indirectness	Imprecision	Heterogeneity	Incoherence	Confidence rating	Reason(s) for downgrading
**Mixed evidence**									
CBT vs Con	3	Some concerns	Some concerns	No concerns	Some concerns	Some concerns	No concerns	Moderate	
CBT vs DBT	1	Some concerns	Some concerns	No concerns	Some concerns	Some concerns	No concerns	Moderate	
CBT vs IR	1	No concerns	Some concerns	No concerns	Some concerns	Some concerns	No concerns	Moderate	
CBT vs RPE	1	No concerns	Some concerns	No concerns	Major concerns	No concerns	No concerns	Low	Imprecision
Con vs DBT	2	Some concerns	Some concerns	No concerns	Major concerns	No concerns	No concerns	Low	Imprecision
Con vs IR	1	No concerns	Some concerns	No concerns	Some concerns	Some concerns	No concerns	Moderate	
Con vs MBE	2	No concerns	Some concerns	No concerns	No concerns	No concerns	No concerns	Moderate	
Con vs PPT	3	Some concerns	Some concerns	No concerns	Some concerns	Some concerns	No concerns	Moderate	
Con vs RPE	3	Some concerns	Some concerns	No concerns	Some concerns	Some concerns	No concerns	Moderate	
Con vs SE	2	No concerns	Some concerns	No concerns	No concerns	Some concerns	No concerns	Moderate	
EBA vs FTE	1	No concerns	Some concerns	No concerns	No concerns	Some concerns	No concerns	Moderate	
EBA vs IR	1	No concerns	Some concerns	No concerns	Some concerns	Some concerns	No concerns	Moderate	
EBA vs SE	1	No concerns	Some concerns	No concerns	Major concerns	No concerns	No concerns	Low	Imprecision
FTE vs IR	2	No concerns	Some concerns	No concerns	Some concerns	Some concerns	No concerns	Moderate	
FTE vs MBE	1	No concerns	Some concerns	No concerns	No concerns	No concerns	No concerns	Moderate	
FTE vs PPT	1	No concerns	Some concerns	No concerns	Major concerns	No concerns	Major concerns	Very low	Imprecision, Incoherence
IR vs PPT	3	Some concerns	Some concerns	No concerns	Some concerns	Some concerns	No concerns	Moderate	
MBE vs SE	1	No concerns	Some concerns	No concerns	No concerns	Some concerns	No concerns	Moderate	
**Indirect evidence**									
CBT vs EBA	--	No concerns	Some concerns	No concerns	Some concerns	Some concerns	Major concerns	Low	Incoherence
CBT vs FTE	--	No concerns	Some concerns	No concerns	Some concerns	Some concerns	Major concerns	Low	Incoherence
CBT vs MBE	--	Some concerns	Some concerns	No concerns	No concerns	Some concerns	Major concerns	Low	Incoherence
CBT vs PPT	--	Some concerns	Some concerns	No concerns	Some concerns	Some concerns	Major concerns	Low	Incoherence
CBT vs SE	--	Some concerns	Some concerns	No concerns	Some concerns	Some concerns	Major concerns	Low	Incoherence
Con vs EBA	--	No concerns	Some concerns	No concerns	No concerns	Some concerns	Major concerns	Low	Incoherence
Con vs FTE	--	No concerns	Some concerns	No concerns	Major concerns	No concerns	Major concerns	Very low	Imprecision, Incoherence
DBT vs EBA	--	Some concerns	Some concerns	No concerns	Some concerns	Some concerns	Major concerns	Low	Incoherence
DBT vs FTE	--	Some concerns	Some concerns	No concerns	Major concerns	No concerns	Major concerns	Very low	Imprecision, Incoherence
DBT vs IR	--	Some concerns	Some concerns	No concerns	Major concerns	No concerns	Major concerns	Very low	Imprecision, Incoherence
DBT vs MBE	--	Some concerns	Some concerns	No concerns	No concerns	No concerns	Major concerns	Low	Incoherence
DBT vs PPT	--	Some concerns	Some concerns	No concerns	Major concerns	No concerns	Major concerns	Very low	Imprecision, Incoherence
DBT vs RPE	--	Some concerns	Some concerns	No concerns	Major concerns	No concerns	Major concerns	Very low	Imprecision, Incoherence
DBT vs SE	--	Some concerns	Some concerns	No concerns	Some concerns	Some concerns	Major concerns	Low	Incoherence
EBA vs MBE	--	No concerns	Some concerns	No concerns	Some concerns	Some concerns	Major concerns	Low	Incoherence
EBA vs PPT	--	No concerns	Some concerns	No concerns	No concerns	Some concerns	Major concerns	Low	Incoherence
EBA vs RPE	--	No concerns	Some concerns	No concerns	Some concerns	Some concerns	Major concerns	Low	Incoherence
FTE vs RPE	--	No concerns	Some concerns	No concerns	Major concerns	No concerns	Major concerns	Very low	Imprecision, Incoherence
FTE vs SE	--	No concerns	Some concerns	No concerns	Some concerns	No concerns	Major concerns	Low	Incoherence
IR vs MBE	--	No concerns	Some concerns	No concerns	No concerns	No concerns	Major concerns	Low	Incoherence
IR vs RPE	--	No concerns	Some concerns	No concerns	Major concerns	No concerns	Major concerns	Very low	Imprecision, Incoherence
IR vs SE	--	No concerns	Some concerns	No concerns	Some concerns	Some concerns	Major concerns	Low	Incoherence
MBE vs PPT	--	No concerns	Some concerns	No concerns	No concerns	No concerns	Major concerns	Low	Incoherence
MBE vs RPE	--	No concerns	Some concerns	No concerns	No concerns	Some concerns	Major concerns	Low	Incoherence
PPT vs RPE	--	Some concerns	Some concerns	No concerns	Some concerns	Some concerns	Major concerns	Low	Incoherence
PPT vs SE	--	Some concerns	Some concerns	No concerns	No concerns	Some concerns	Major concerns	Low	Incoherence
RPE vs SE	--	Some concerns	Some concerns	No concerns	Some concerns	Some concerns	Major concerns	Low	Incoherence

Figure S3 CINeMA Ratings: Anxiety assessed at the comparison average ROB and indirectness
Note: CBT: Cognitive Behavioral Therapy; EBA: Education with Behavioral Activation; RPE: Relaxation and Psychological Education; MBE: Mind Body Exercise; SE: Structured Exercise; FTE: Functional and Targeted Exercise; PPT: Passive Physical Therapy; DBT: Digital and Biofeedback Therapy; IR: Integrated Rehabilitation.

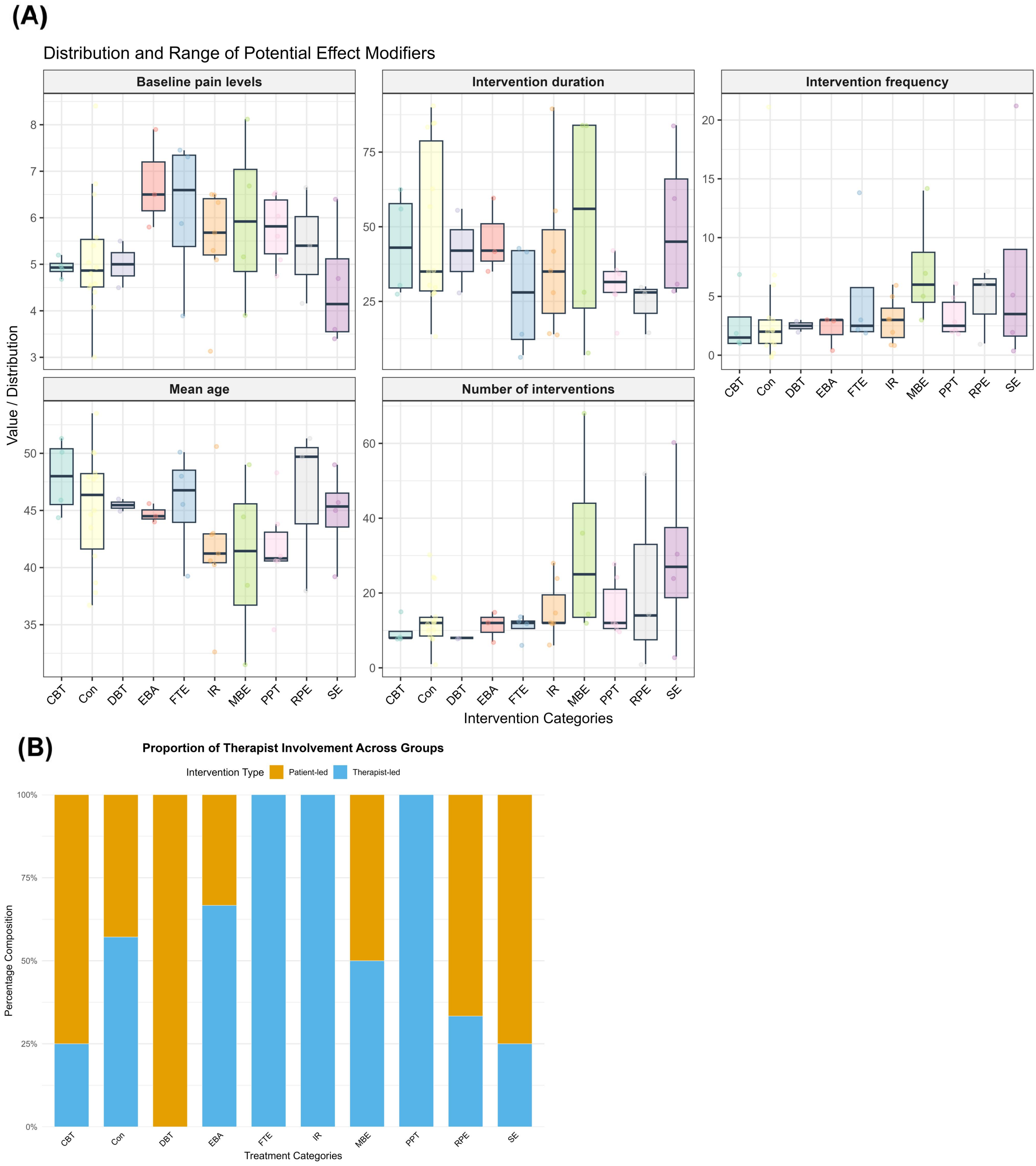

Figure S4 (A) Box-and-whisker plot for continuous variables; (B) Bar chart showing percentages. **Note:** (A) The box represents the interquartile range; the thick horizontal line in the middle indicates the median; the whiskers at either end indicate the range of values; the coloured data points represent the specific values from each original study. CBT: Cognitive Behavioral Therapy; EBA: Education with Behavioral Activation; RPE: Relaxation and Psychological Education; MBE: Mind Body Exercise; SE: Structured Exercise; FTE: Functional and Targeted Exercise; PPT: Passive Physical Therapy; DBT: Digital and Biofeedback Therapy; IR: Integrated Rehabilitation.

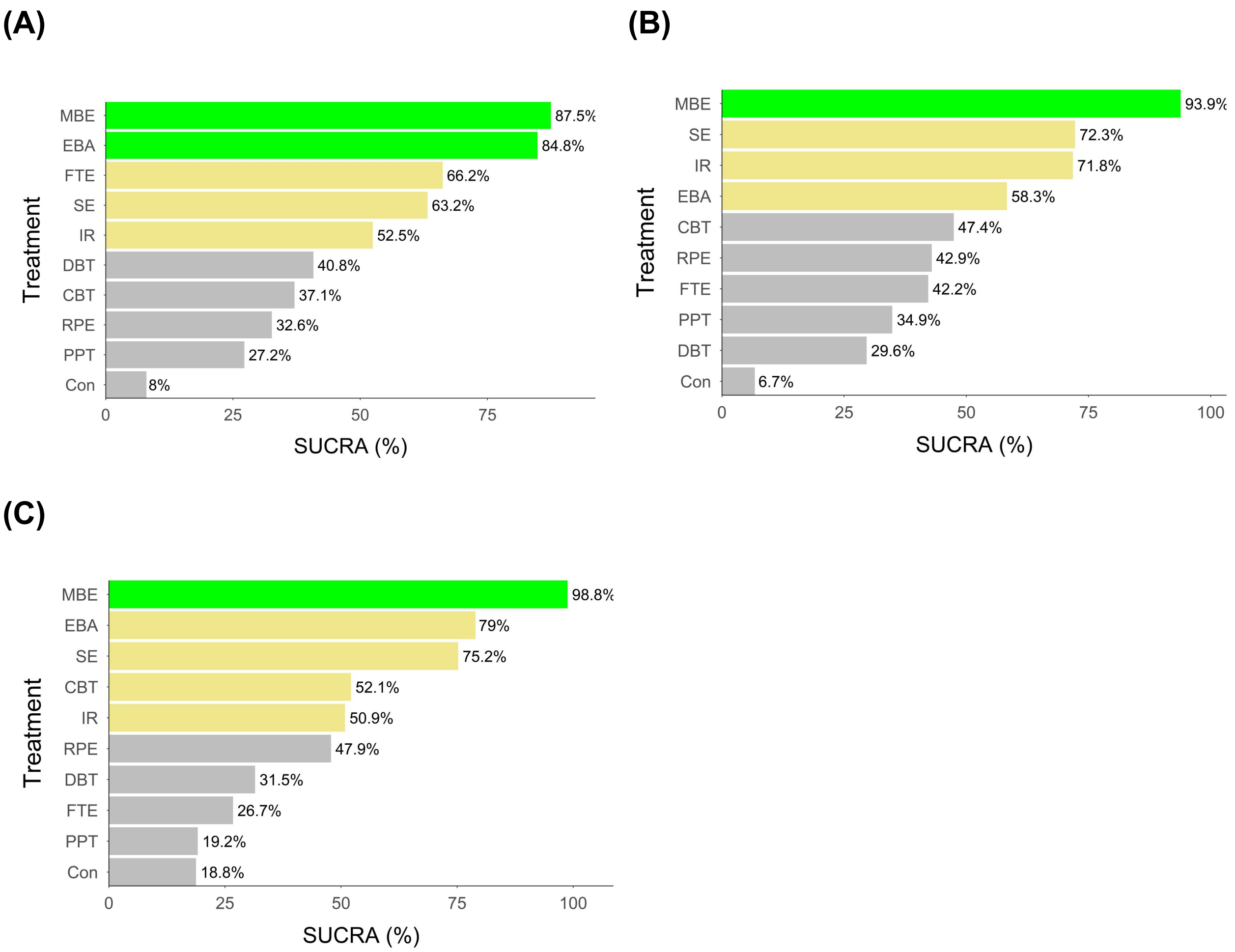

Figure S5 Ranked bar chart of (A) Pain; (B) Depression; (C) Anxiety.

Note: Interventions are ordered from highest to lowest SUCRA, and bars are coloured by SUCRA level: green ≥ 80% (high), khaki 50–80% (medium), grey < 50% (low). CBT: Cognitive Behavioral Therapy; EBA: Education with Behavioral Activation; RPE: Relaxation and Psychological Education; MBE: Mind Body Exercise; SE: Structured Exercise; FTE: Functional and Targeted Exercise; PPT: Passive Physical Therapy; DBT: Digital and Biofeedback Therapy; IR: Integrated Rehabilitation.

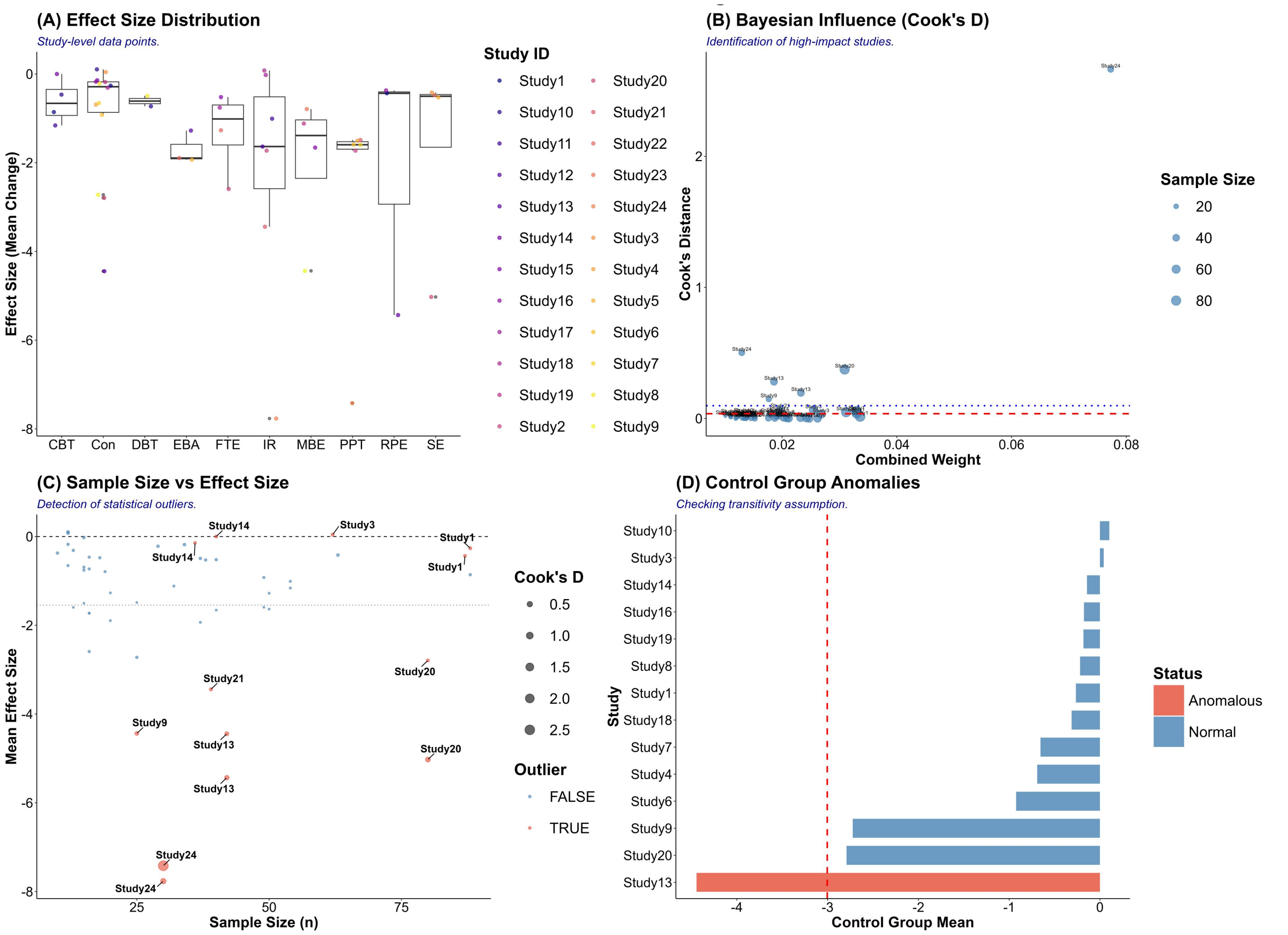

Figure S6 Diagnostic plots for pain : (A) Effect Size Distribution, (B) Bayesian Influence (Cook's D), (C) Sample Size vs Effect Size, (D) Control Group Anomalies.

Note: (A) Box plots showing the distribution of effect sizes for each intervention and a scatter plot identifying potential outlier studies; (B) the red dashed line represents the 75th percentile, and studies above this line are considered to have a significant influence on the model estimates. (C) Weighted diagnostic plot; the red markers indicate statistical outliers, i.e. cases where the observed effect size deviates significantly from the network's expected precision-weighted mean. (D) The red bars indicate studies in which the control group response deviated by more than 1.5 standard deviations from the network baseline mean. CBT: Cognitive Behavioral Therapy; EBA: Education with Behavioral Activation; RPE: Relaxation and Psychological Education; MBE: Mind Body Exercise; SE: Structured Exercise; FTE: Functional and Targeted Exercise; PPT: Passive Physical Therapy; DBT: Digital and Biofeedback Therapy; IR: Integrated Rehabilitation.

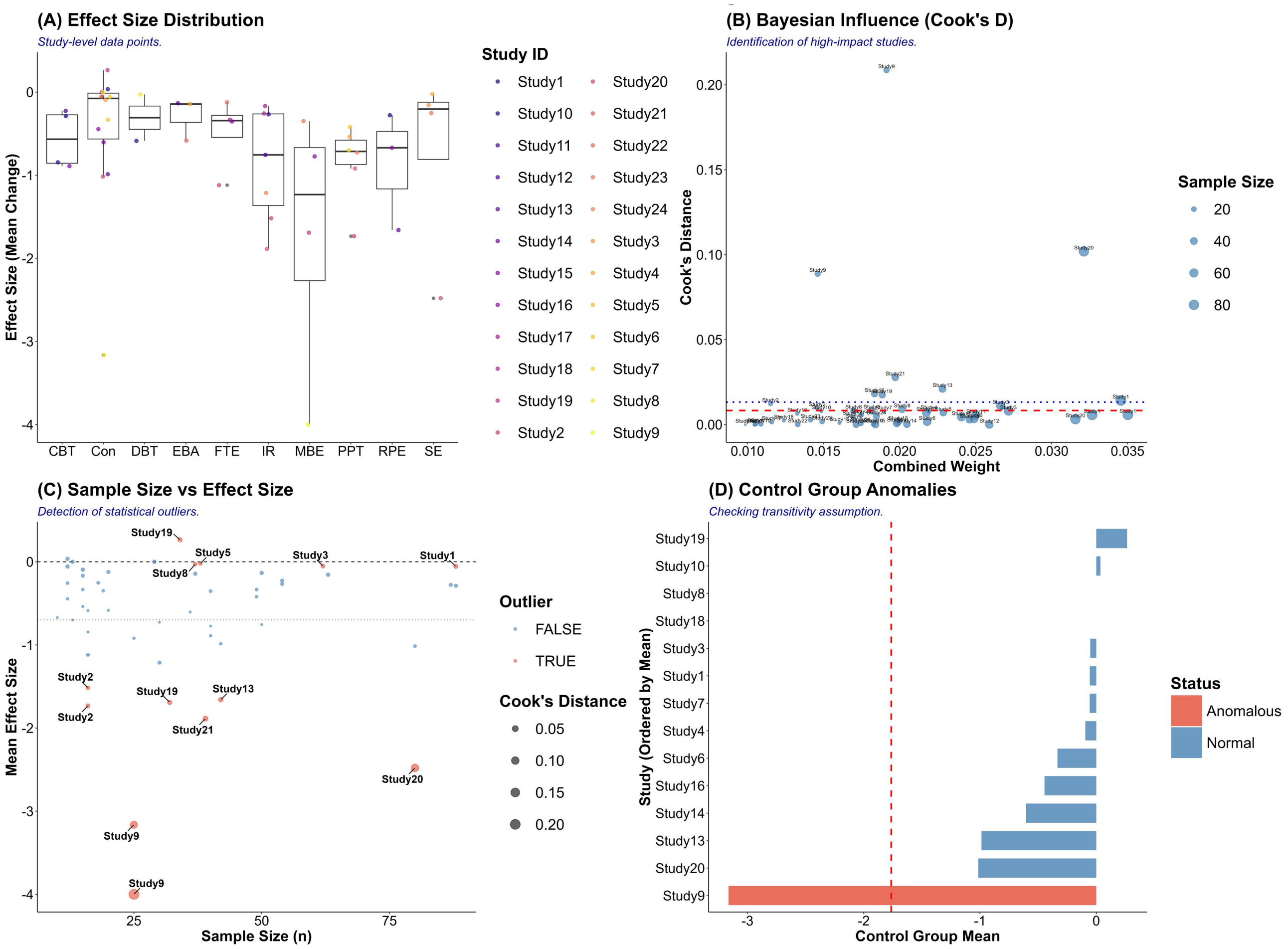

Figure S7 Diagnostic plots for depression: (A) Effect Size Distribution, (B) Bayesian Influence (Cook's D), (C) Sample Size vs Effect Size, (D) Control Group Anomalies.

Note: (A) Box plots showing the distribution of effect sizes for each intervention and a scatter plot identifying potential outlier studies; (B) the red dashed line represents the 75th percentile, and studies above this line are considered to have a significant influence on the model estimates. (C) Weighted diagnostic plot; the red markers indicate statistical outliers, i.e. cases where the observed effect size deviates significantly from the network’s expected precision-weighted mean. (D) The red bars indicate studies in which the control group response deviated by more than 1.5 standard deviations from the network baseline mean. CBT: Cognitive Behavioral Therapy; EBA: Education with Behavioral Activation; RPE: Relaxation and Psychological Education; MBE: Mind Body Exercise; SE: Structured Exercise; FTE: Functional and Targeted Exercise; PPT: Passive Physical Therapy; DBT: Digital and Biofeedback Therapy; IR: Integrated Rehabilitation.

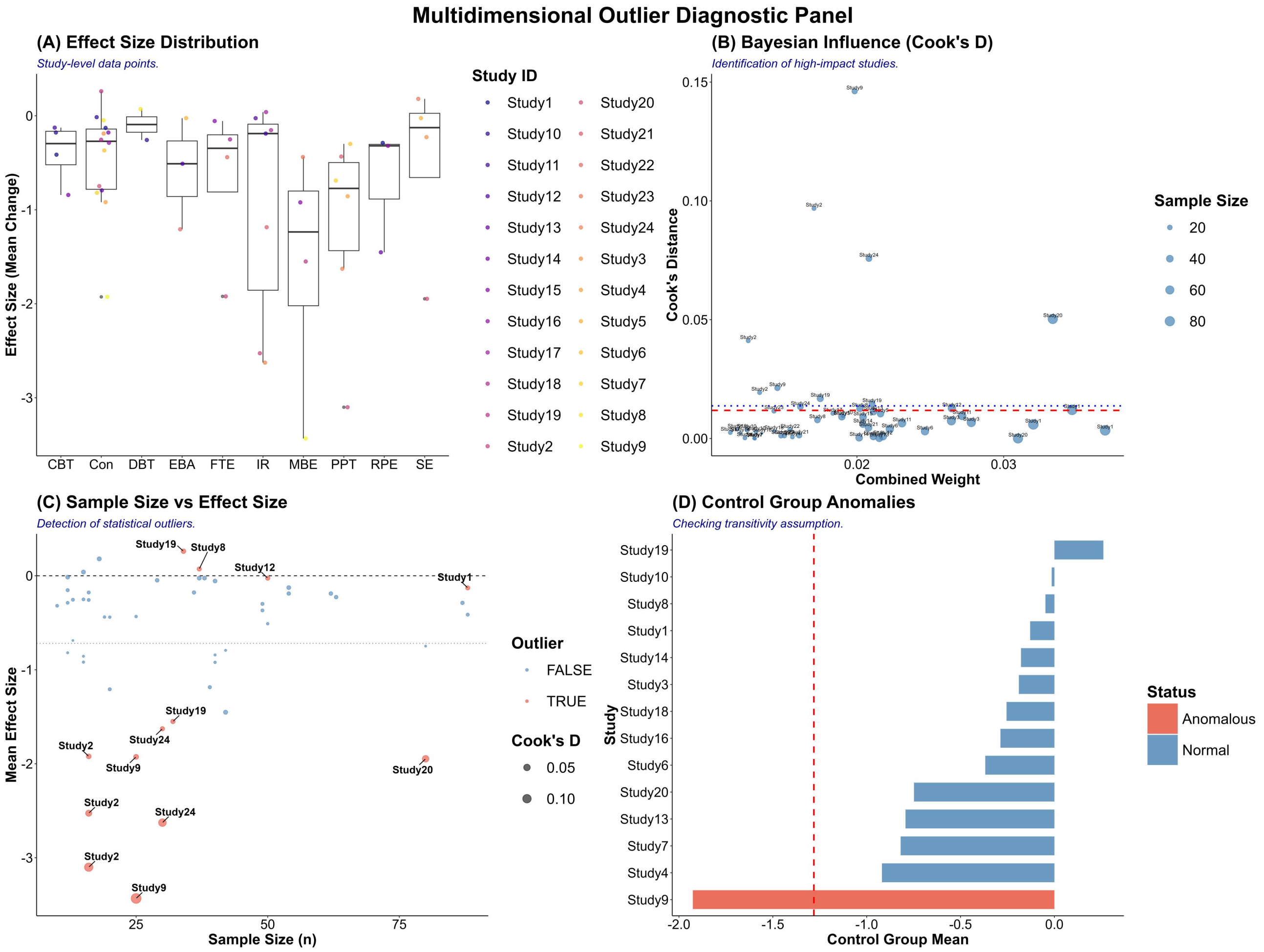

Figure S8 Diagnostic plots for anxiety: (A) Effect Size Distribution, (B) Bayesian Influence (Cook's D), (C) Sample Size vs Effect Size, (D) Control Group Anomalies.

Note: (A) Box plots showing the distribution of effect sizes for each intervention and a scatter plot identifying potential outlier studies; (B) the red dashed line represents the 75th percentile, and studies above this line are considered to have a significant influence on the model estimates. (C) Weighted diagnostic plot; the red markers indicate statistical outliers, i.e. cases where the observed effect size deviates significantly from the network’s expected precision-weighted mean. (D) The red bars indicate studies in which the control group response deviated by more than 1.5 standard deviations from the network baseline mean. CBT: Cognitive Behavioral Therapy; EBA: Education with Behavioral Activation; RPE: Relaxation and Psychological Education; MBE: Mind Body Exercise; SE: Structured Exercise; FTE: Functional and Targeted Exercise; PPT: Passive Physical Therapy; DBT: Digital and Biofeedback Therapy; IR: Integrated Rehabilitation.

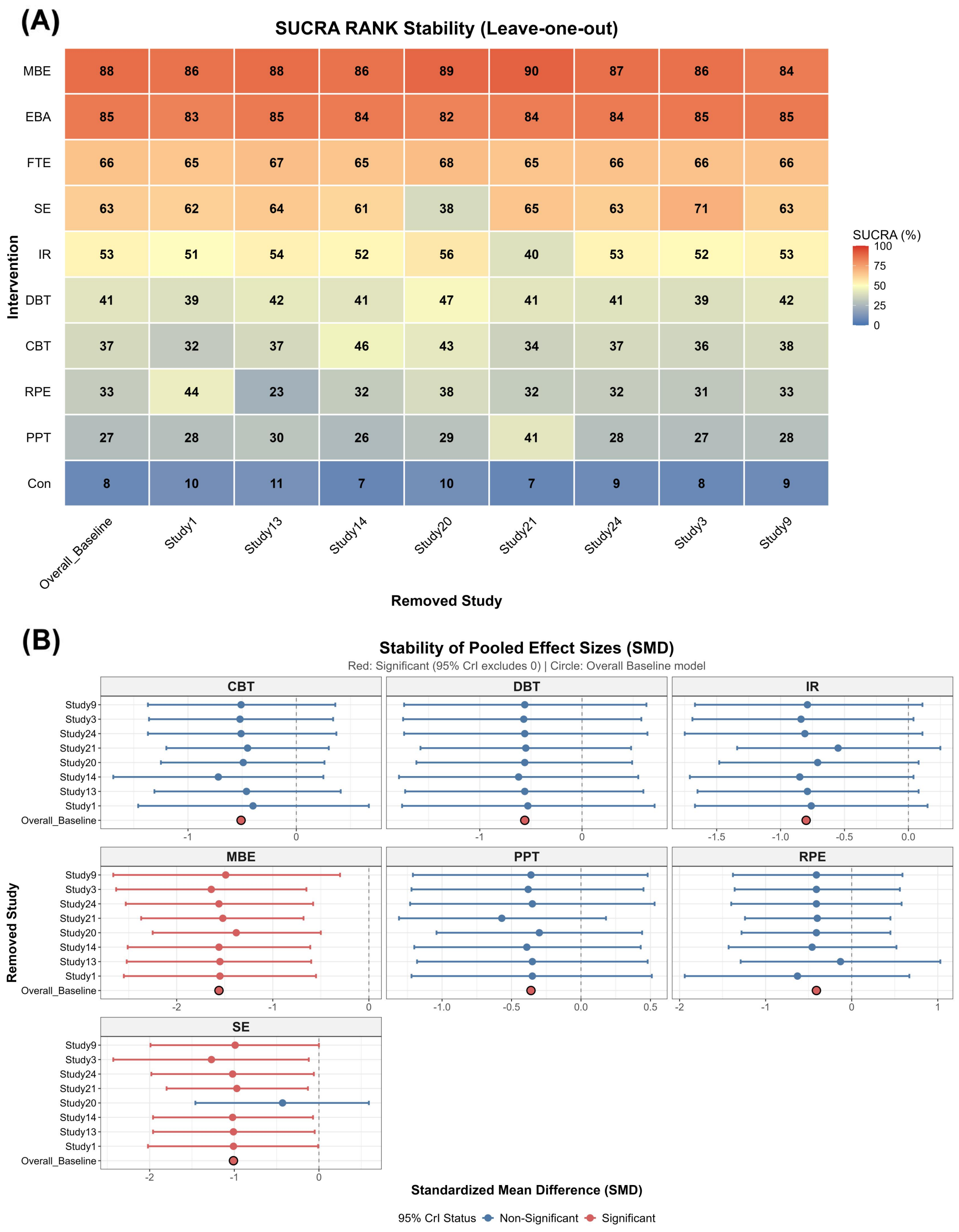

Figure S9 Sensitivity analysis of pain: (A) SUCRA Rank Stability, (B) Stability of Pooled Effect Sizes.

Note: (A) The numbers within the cells represent SUCRA values (%), with colours ranging from blue to red indicating efficacy ranging from poor to excellent.; (B) Red indicates that the 95% confidence interval (95% CrI) does not include 0 (statistically significant), whilst blue indicates no significance.. CBT: Cognitive Behavioral Therapy; EBA: Education with Behavioral Activation; RPE: Relaxation and Psychological Education; MBE: Mind Body Exercise; SE: Structured Exercise; FTE: Functional and Targeted Exercise; PPT: Passive Physical Therapy; DBT: Digital and Biofeedback Therapy; IR: Integrated Rehabilitation.

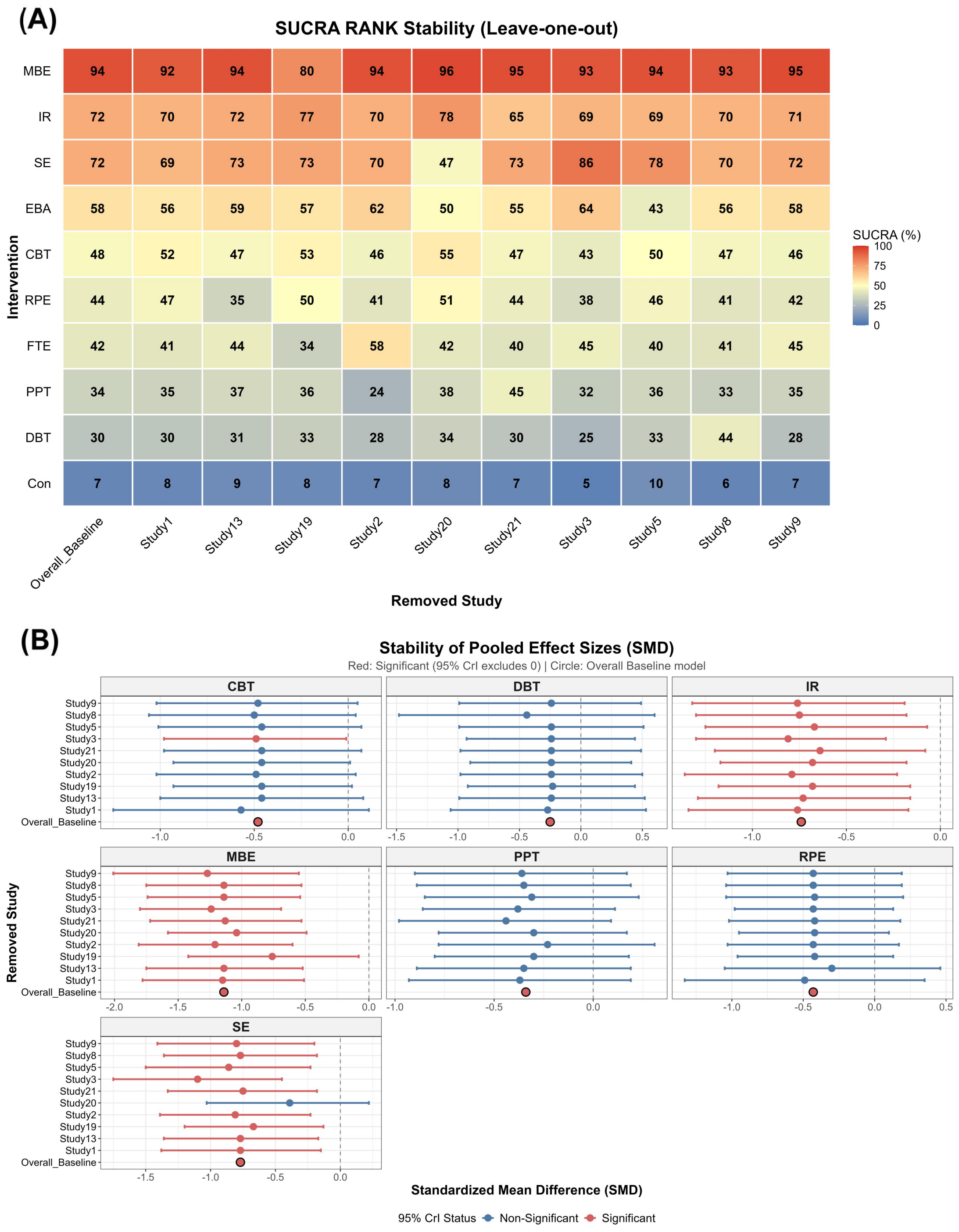

Figure S10 Sensitivity analysis of depression: (A) SUCRA Rank Stability, (B) Stability of Pooled Effect Sizes.

Note: (A) The numbers within the cells represent SUCRA values (%), with colours ranging from blue to red indicating efficacy ranging from poor to excellent.; (B) Red indicates that the 95% confidence interval (95% CrI) does not include 0 (statistically significant), whilst blue indicates no significance.. CBT: Cognitive Behavioral Therapy; EBA: Education with Behavioral Activation; RPE: Relaxation and Psychological Education; MBE: Mind Body Exercise; SE: Structured Exercise; FTE: Functional and Targeted Exercise; PPT: Passive Physical Therapy; DBT: Digital and Biofeedback Therapy; IR: Integrated Rehabilitation.

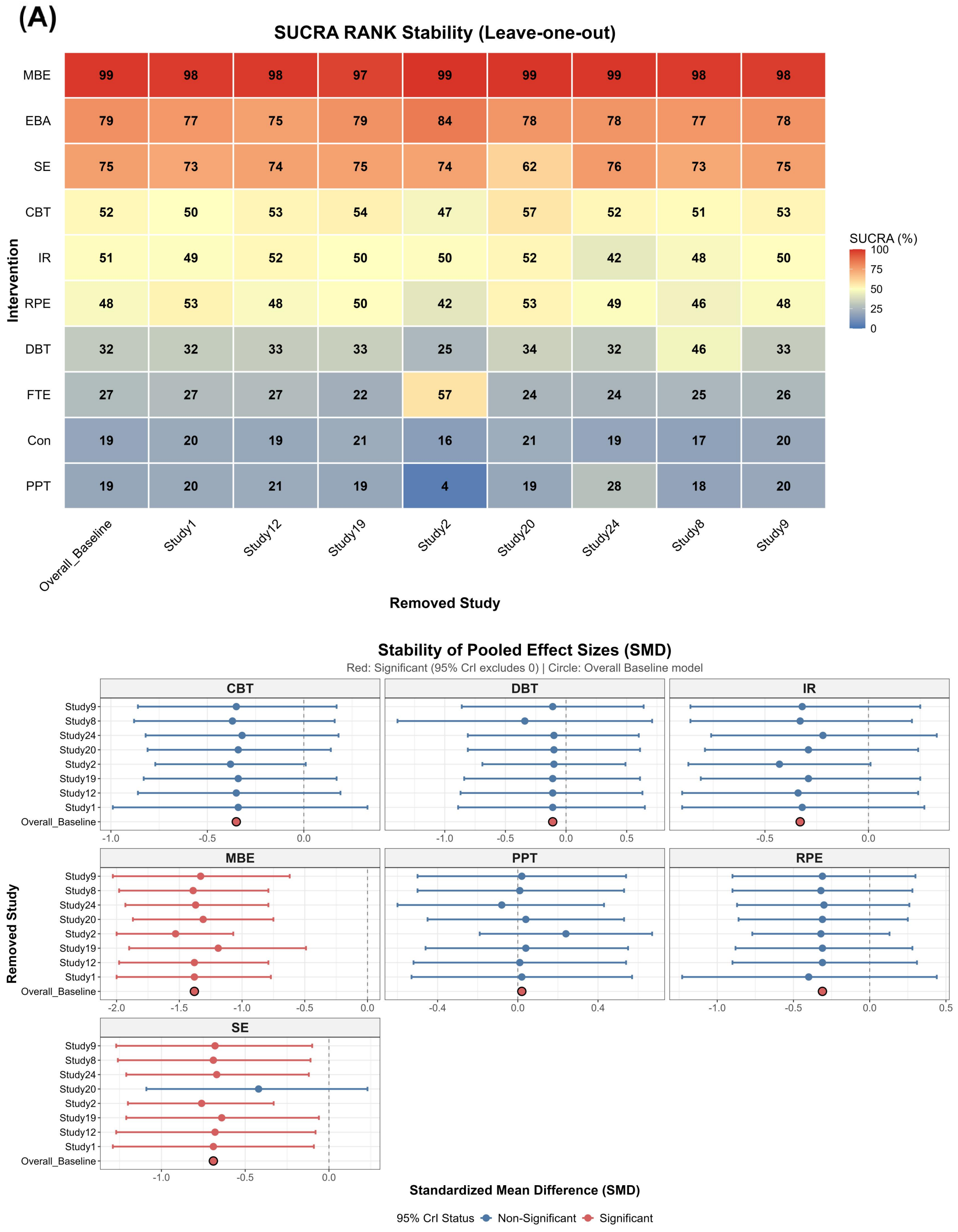

Figure S11 Sensitivity analysis of anxiety: (A) SUCRA Rank Stability, (B) Stability of Pooled Effect Sizes.

Note: (A) The numbers within the cells represent SUCRA values (%), with colours ranging from blue to red indicating efficacy ranging from poor to excellent.; (B) Red indicates that the 95% confidence interval (95% CrI) does not include 0 (statistically significant), whilst blue indicates no significance.. CBT: Cognitive Behavioral Therapy; EBA: Education with Behavioral Activation; RPE: Relaxation and Psychological Education; MBE: Mind Body Exercise; SE: Structured Exercise; FTE: Functional and Targeted Exercise; PPT: Passive Physical Therapy; DBT: Digital and Biofeedback Therapy; IR: Integrated Rehabilitation.

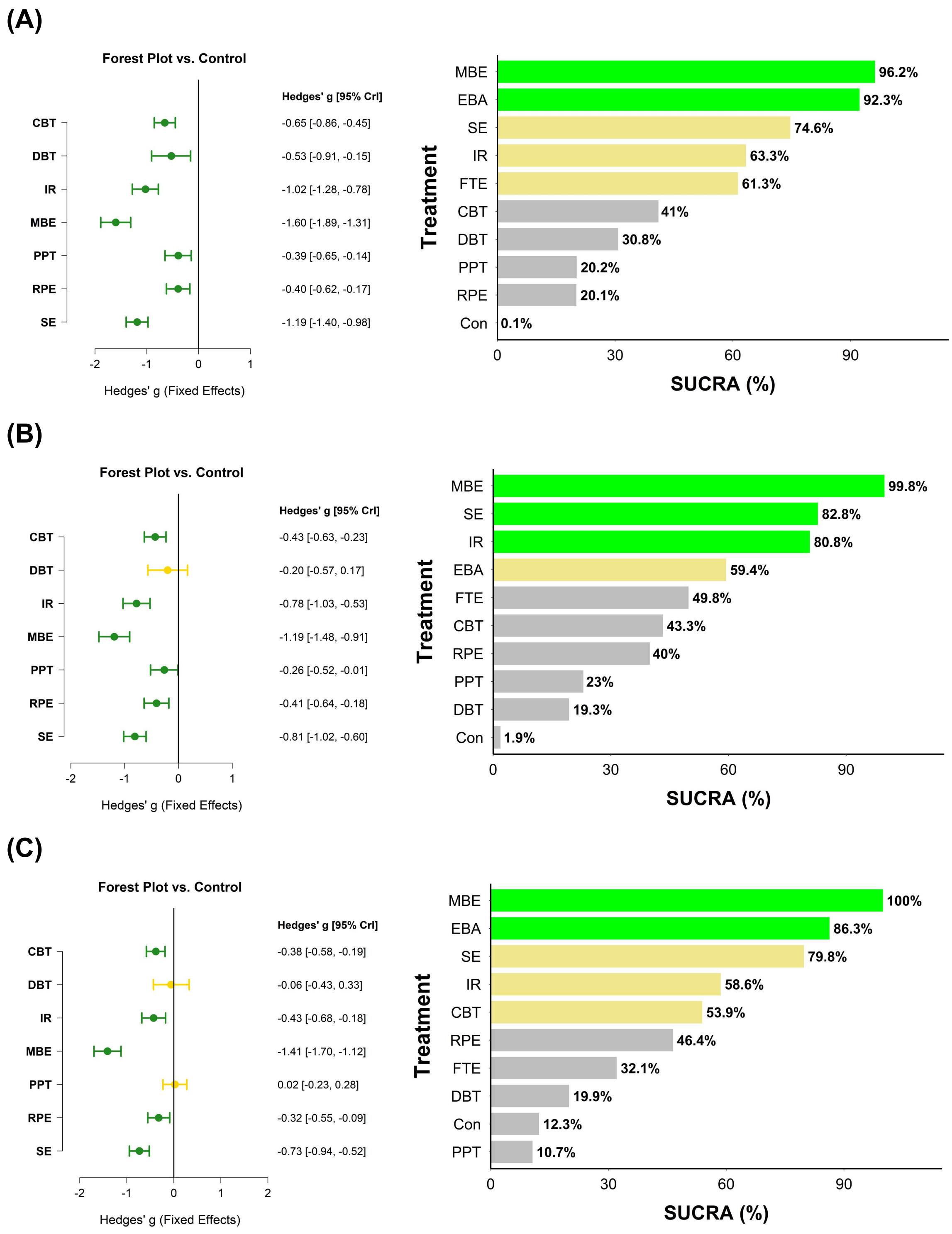

Figure S12 NMA forest plot and Ranked bar chart of (A) Pain; (B) Depression; (C) Anxiety.

Note: Colours in the left-hand figure reflect the posterior probability of direction: green = beneficial (95 % CrI entirely below zero), gold = no clear difference (95 % CrI spans zero), red = harmful (95 % CrI entirely above zero). The right-hand figure is coloured by SUCRA level: green ≥ 80% (high), khaki 50–80% (medium), grey < 50% (low). CBT: Cognitive Behavioral Therapy; EBA: Education with Behavioral Activation; RPE: Relaxation and Psychological Education; MBE: Mind Body Exercise; SE: Structured Exercise; FTE: Functional and Targeted Exercise; PPT: Passive Physical Therapy; DBT: Digital and Biofeedback Therapy; IR: Integrated Rehabilitation.
